# Highly mismatch-tolerant homology testing by RecA could explain how homology length affects recombination

**DOI:** 10.1371/journal.pone.0288611

**Published:** 2023-07-13

**Authors:** Mara Prentiss, Dianzhuo Wang, Jonathan Fu, Chantal Prévost, Veronica Godoy-Carter, Nancy Kleckner, Claudia Danilowicz

**Affiliations:** 1 Department of Physics, Harvard University, Cambridge, Massachusetts, United States of America; 2 Laboratoire de Biochimie Théorique, Institut de Biologie Physico-Chimique, Paris, France; 3 Department of Biology, Northeastern University, Boston, Massachusetts, United States of America; 4 Department of Molecular and Cellular Biology, Harvard University, Cambridge, Massachusetts, United States of America; Tulane University Health Sciences Center, UNITED STATES

## Abstract

In *E*. *coli*, double strand breaks (DSBs) are resected and loaded with RecA protein. The genome is then rapidly searched for a sequence that is homologous to the DNA flanking the DSB. Mismatches in homologous partners are rare, suggesting that RecA should rapidly reject mismatched recombination products; however, this is not the case. Decades of work have shown that long lasting recombination products can include many mismatches. In this work, we show that *in vitro* RecA forms readily observable recombination products when 16% of the bases in the product are mismatched. We also consider various theoretical models of mismatch-tolerant homology testing. The models test homology by comparing the sequences of L_test_ bases in two single-stranded DNAs (ssDNA) from the same genome. If the two sequences pass the homology test, the pairing between the two ssDNA becomes permanent. Stringency is the fraction of permanent pairings that join ssDNA from the same positions in the genome. We applied the models to both randomly generated genomes and bacterial genomes. For both randomly generated genomes and bacterial genomes, the models show that if no mismatches are accepted stringency is ∼ 99% when L_test_ = 14 bp. For randomly generated genomes, stringency decreases with increasing mismatch tolerance, and stringency improves with increasing L_test_. In contrast, in bacterial genomes when L_test_ ∼ 75 bp, stringency is ∼ 99% for both mismatch-intolerant and mismatch-tolerant homology testing. Furthermore, increasing L_test_ does not improve stringency because most incorrect pairings join different copies of repeats. In sum, for bacterial genomes highly mismatch tolerant homology testing of 75 bp provides the same stringency as homology testing that rejects all mismatches and testing more than ∼75 base pairs is not useful. Interestingly, *in vivo* commitment to recombination typically requires homology testing of ∼ 75 bp, consistent with highly mismatch intolerant testing.

## Introduction

RecA-family proteins occur in all domains of life [1]. RecA-family proteins promote repair of double strand breaks (DSB) in genomes [1–3]. Incorrect repair of double-strand breaks can cause cancer [4] and birth defects [4]. Thus, genome integrity should be maintained; however, genomic rearrangements [5, 6] can be highly advantageous. An example is bacterial acquisition of antibiotic resistances [7]. As a result, it is critical to understand how RecA-family proteins repair double-strand breaks and how that repair affects the balance between genome integrity and introducing rare and perhaps highly advantageous genomic rearrangements.

After a double strand break, resection of the broken double-stranded DNA (dsDNA) creates 3′-ssDNA tails [2, 8]. Those ssDNA tails are often referred to as invading strands. After an invading strand is covered by RecA protein, each ssDNA-RecA filament probes an intact copy of the broken chromosome. The sequence of the unbroken chromosome is probed by attempting to establish Watson-Crick pairing between the invading strand and one of the strands in the target thus forming a heteroduplex product and leaving the other strand in the target unpaired [2, 8]. The resulting structure is called a D-loop.

*In vivo* studies probed the homology dependence of recombination using exogenous sequences containing L contiguous base pairs that were homologous to the target DNA [9–11]. The homologous bases were flanked by extensive heterology. Those *in vivo* studies found that when L < 20 bp, recombination was not detectable. A steep exponential increase in recombination with length was observed for 20 ⪅ L ⪅ 75 bp [9, 10], and a slow linear increase in recombination was observed for L ⪆ 75 bp [10]. Thus, *in vivo* studies suggest that the behavior of recombination products as a function of L divides into three regions. The boundaries between the regimes occur at ∼ 20 bp and ∼ 75 bp. Furthermore, the *in vivo* studies suggest that testing more than 75 bp may not be very useful.

*In vivo* experiments have also considered how periodically spaced mismatches in otherwise homologous invading strands influence homologous recombination in yeast [12]. That work found that when the invading strand included 1 mismatch/6 bases repair was ∼ 5% efficient, whereas more frequent mismatch spacings did not produce observable repair. Furthermore, *in vitro* studies have found that RecA mediated homologous recombination is very tolerant of mismatches [13–17].

Though the detailed mechanisms underlying RecA mediated homologous recombination are not fully understood, it is believed that homology testing by RecA starts with the initial 8 bp homology test [16, 18–21] that accepts one mismatch [18]. That initial test is followed by homology testing that groups base pairs into triplets. Each triplet is tested for homology separately. For each triplet, the outcome of the homology test depends on the number of mismatches within the triplet [22]. Such a division of homology testing into tests of separate base pair triplets is consistent with the known structure of heteroduplex products. The crystal structure indicates that heteroduplex products are divided into nearly B-form base pair triplets that are separated by large rises [23]. The triplets are stabilized by protein residues that intercalate in the rises [18, 23].

It has long been known that RecA mediated homologous recombination can occur without ATP hydrolysis [24]. Some features of homology recognition are independent of hydrolysis. For example, the initial 8 bp test occurs both with [16] and without hydrolysis [18–21]; however, it is likely that some RecA features depend on hydrolysis. Importantly, without ATP hydrolysis, heteroduplex products longer than ∼ 20 bp are effectively irreversible [24], whereas with ATP hydrolysis 20 bp products remain highly reversible *in vitro* [25] and *in vivo* [9–11]. Thus, the reversal of heteroduplex products longer than 20 bp is clearly strongly influenced by ATP hydrolysis.

In this work we probe RecA strand exchange *in vitro* with ATP hydrolysis. Consistent with previous work in yeast [12], we show that if mismatches between the invading and complementary strands are distributed periodically, 1 mismatch/3 bases blocks formation of stable strand exchange products, whereas 1 mismatch/6 bases forms readily observable heteroduplex products. Given that homology-testing *in vitro* suggests that RecA family protein mediated strand exchange is very tolerant of mismatches, we use simple theoretical models to probe highly mismatch-tolerant homology testing. In all the theoretical models, two sequences containing L_test_ base pairs are tested for homology. If the two sequences pass the homology test, the pairing between the two sequences becomes stable. That stable pairing creates a heteroduplex product. We then consider the fraction of those heteroduplex products that correctly pair corresponding sequence regions in two chromosomes. When we apply homology testing to a “genome” consisting of randomly chosen bases, the fraction of incorrect pairings that passes the homology test always decreases as L_test_ increases. In contrast, applying the same homology tests to bacterial genomes indicates that when L_test_ > 75 bp, the fraction of incorrect pairings that passes the homology test does not decrease with increasing L_test_ because most incorrect products join different copies of sequences that are exactly repeated in the genome. Importantly, even if our models do not accurately reflect all features of RecA strand exchange *in vivo*, insights from robust features of the modeling results may greatly enhance our understanding of RecA mediated homology recognition.

## Materials and methods

### Sample preparation

The 180 bp rhodamine and fluorescein labelled dsDNA was prepared by initially annealing an internal rhodamine 90-nt ssDNA (O1) and a 5´end phosphorylated oligonucleotide (82 bases) (O2). Similarly, an 82-nt 5´-end phosphorylated oligonucleotide (O3) was annealed with a 98-nt oligonucleotide containing an internal fluorescein label (O4) to obtain another labelled dsDNA fragment. These two dsDNA labelled fragments were annealed and ligated overnight at 16°C with T4 DNA ligase in ligase reaction buffer (50 mM Tris, 10 mM MgCl_2_, 1 mM ATP, and 10 mM dithiothreitol, pH 7.5 (New England Biolabs (NEB)). The 180 bp construct was purified3 by gel on 3% agarose in TBE (Tris/Borate/EDTA) buffer for 2 hours (6 V/cm). The 180 bp band was visualized with a midrange UV trans-illuminator. Finally, the 180 bp dsDNA band was cut out and was extracted from agarose using a Nucleospin kit (Machery and Nagel, Bethlehem, PA). The product was finally concentrated using 100 kDa Amicon filters (Millipore). The oligonucleotide sequences (Integrated DNA Technologies (Coralville, IA)) used to prepare the dsDNA construct are listed in S1 Table.

### Strand exchange reactions

DNA strand exchange *in vitro* was achieved by mixing 0.06 μM ssDNA-RecA filaments with 0.06 μM labeled dsDNA. The sequences of the oligonucleotides used for these filaments are listed in S1 Table. The ssDNA-RecA filaments were prepared by mixing the ssDNA (final concentration 6 μM in bases) with 2 μM RecA (NEB) at 3:1 ratio (bases: protein), 1 mM ATP, a regeneration system containing 10 U/ml of pyruvate kinase and 3 mM phosphoenolpyruvate, and 0.2 μM *E*.*coli* single-stranded binding protein, SSB (Abcam) in RecA buffer (70 mM Tris-HCl, 10 mM MgCl_2_, and 5 mM dithiothreitol, pH 7.6). Filament formation proceeded at 37°C for 10 minutes.

### Fluorescence measurements

To measure the fluorescent signal of these strand exchange reactions, the ssDNA/RecA filament and dsDNA mixture was placed in a quartz cuvette. Initially, fluorescein emission was quenched by rhodamine because the outgoing and complementary strands were paired in the dsDNA. As strand exchange progresses, the outgoing and complementary strands separate and fluorescein emission increases. Using FluorEssence spectroscopy software and the automated FluoroMax spectrofluorometer (Horiba, Edison, NJ), the emission of the fluorescein label was read using 493 nm excitation wavelength and a 2 nm slit. The emission of the fluorescein fluorophore in counts per second (cps) was detected at 518 nm with a 2 nm slit. The reaction was run for 30 minutes, and emission measurements were collected every 1 second with an integration time of 0.5 s. Temperature was kept constant at 37°C.

### Probabilities of incorrect pairings for a homology search following a DSB in a random genome: Analytical approach

For a random genome, the probability that one randomly chosen base will match another randomly chosen base is p = ¼. The probability of finding m or more correctly paired bases in a randomly chosen sample of n base pairs is then:

$$P(m,\;n)\;=\;\sum_{k=m}^{n}\left(\begin{array}{l} n\\ k \end{array}\right)p^{k}{\left(1-p\right)}^{n-k}$$ (1)

For the 8 bp test, n = 8 and for the triplet tests n = 3. In either case m is the difference between n and the number of mismatches.

Thus, for an 8 bp test with one mismatch n = 8 and m = 7, and for a triplet test that accepts two mismatches n = 3 and m = 1.

The probability of passing a series of tests is just the product of the probabilities for all of the tests. Thus, for a given L_test_, the probability of passing an 8 bp test followed by a series of triplet tests is the product of the probability of passing the first 8 bp test times the probability of passing (L_test_-8)/3 triplet tests, where N_mismatch_ and N_mismatch8_ are the number of mismatches accepted per triplet and 8 bp test, respectively.


$$\text{Pass}\left({\text{L}}_{\text{test}},{\text{N}}_{\text{mismatch,}},\;{\text{N}}_{\text{mismatch8}}\right)\;=\;\text{P}\left(8-{\text{N}}_{\text{mismatch8}},8\right)\;\text{P}{\left(3-{\text{N}}_{\text{mismatch}},\;3\right)}^{(\text{Ltest-8)/3}}$$ (2)

The formula implies that the results for a series of triplet tests that accept only one mismatch per triplet are much more stringent than the results of a test that accepts 1/3 mismatches in L_test_-8 bases.

For a genome that includes L_genome_ randomly chosen bases, and a randomly chosen sequence of length L_test_, in the genome there are on average Pass(L_test_, N_mismatch_, N_mismatch8_) L_genome_ matching sequences of length L_test_.

In DSB repair for a random genome, we assume that the sequence of the searcher is always included in the target; therefore, there is also always one correct pairing between the searcher and the target, regardless of the probability that a randomly chosen searching sequence would find a match. Thus, for a random genome during the homology search that follows a DSB, the average number of pairings that would pass the RecA homology test is:

$$\begin{array}{c} {\text{Number}}_{\text{pass}}\left({\text{N}}_{\text{mismatch}},\;{\text{N}}_{\text{mismatch}}\text{,}\;{\text{L}}_{\text{test}},\;{\text{L}}_{\text{genome}\;}\right)\\ =1\;+\;\text{Pass}\left({\text{L}}_{\text{teste}},\;{\text{N}}_{\text{mismatch}\;},\;{\text{N}}_{\text{mismatch8}}\right)\;{\text{L}}_{\text{genome}\;} \end{array}$$ (3)

Of those that pass, probability of being correct is then 1 out of the total number or

$${\text{Prob}}_{\text{pass}}\left({\text{N}}_{\text{mismatck},}\;{\text{N}}_{\text{mismatch8},}\;{\text{L}}_{\text{test}},\;{\text{L}}_{\text{genome}\;}\right)\;=\;1/\left(1\;+\;\text{Pass}\left(L_{\text{test}},\;N_{\text{mismatch}},\;N_{\text{mismatch}8}\right)L_{\text{genome}}\right)$$ (4)

The probability that a pairing passes and is incorrect is then:

$${\text{Prob}}_{\text{incorrect}}\left({\text{N}}_{\text{mismatch}},\;{\text{N}}_{\text{mismatch8,}\;}\;{\text{L}}_{\text{test}},\;{\text{L}}_{\text{genome}\;}\right)\;\;=\;1/\left(1\;+\;\text{Pass}\left(L_{\text{test}},\;N_{\text{mismatch}},\;N_{\text{mismatch8}}\right)L_{\text{genome}}\right)$$ (5)

We note that all predictions of the analytical treatments are independent of the following: 1. The directionality of strand exchange; 2. whether strand exchange is unidirectional or bidirectional; 3. whether the bases are tested iteratively or all at once.

### Probabilities of incorrect pairings for a bacterial genome using the simplified model that is applied sparsely to the sequences of bacterial genomes or a random genome: Simulation approach

A position in the given strand of genome was randomly chosen as the position of the 5′ end of the invading strand. A second position in the given strand of the genome was randomly chosen to represent the testing position in an unbroken chromosome that pairs with the 5′ end of the invading strand. The sequences of the 8 bp starting at the 5′ end and extending in the 3′ direction were then compared. If the number of mismatches was > N_mismatch8_, homology testing at that position terminated and a new testing position was chosen. If the number of mismatches was ≤ N_mismatch8_, then the next 3 bases on the 3′ side were tested for homology. Homology testing terminates, and a new testing position is chosen whenever the number of mismatches in a triplet > N_mismatch_. If the length that has passed the homology tests reaches L_test_, then the pairing is considered irreversible. If the test position and the search position are the same, the irreversible pairing is correct. If the test position and search position are different but there are no mismatches, then the irreversible pairing joins two copies of a repeat with length ≥ L_test_. If the irreversible pairing contains mismatches, the pairing is an error. Typically, 100–100000 different invading strand sequences were chosen, and the results represent the averages of the results for the chosen invading strand sequences out of 4.6 Mbp possible invading strand sequences.

The computer simulations only included ∼ 100 to 10000 simulated DSB positions, so the genome was sparsely sampled; however, we increased the number of simulated breaks until results were insensitive to the number of simulated breaks. In addition, when no mismatches are accepted it is possible to sample the entire genome, and results for sampling the entire genome are similar to results for sparse sampling. We note that results for sampling the entire genome and rejecting all mismatches are independent of the directionality of strand exchange and are not affected by whether strand exchange is unidirectional or bidirectional. Finally, we also applied the computer simulation to random genomes, and the results of the computer simulations are in good agreement with the analytical results for sequences whose bases are chosen at random (random genomes).

We note that the results of these simulations are independent of whether the testing occurs simultaneously or iteratively. The model does assume that the triplet tests occur on the 3′ side of the 8 bp test, but results are insensitive to the positioning of the 8 bp test with respect to the triplet tests. Thus, bidirectional homology testing or 3′ to 5′ homology testing would give very similar results. Finally, exact homology testing that rejects all mismatches is independent of strand exchange directionality, and exact homology testing predicts the same stringency vs. L_test_ as homology testing in which the triplets are positioned to the 3′ side of the initial 8 bp test.

### Exact probabilities of incorrect pairings when N_mismatch_ = 0 for entire bacterial genomes

Each possible 8 bp sequence was assigned a unique mapping number. The bacterial genome was divided into 8 bp sequences, each of which was assigned the corresponding mapping number. The 8 bp sequences were then sorted according to their mapping number, which grouped the entire genome into distinct 8 bp repeats. The total number of incorrect pairing locations for each member of the group is equal to the number of locations in the group -1. We repeated the same procedure for 11 bp sequences. To calculate exact repeats for 14, 17, 22, 33, 44, 55, and 99 bp we created a list of maps encoding a series of sequences with lengths ≤ 11 bp. For example, for L_test_ = 14 we used an 11 bp map and a 3 bp map and then grouped starting locations with the same values for both maps. For the *E*.*coli* MG1655 genome most 99 bp repeats occurred only twice, but some repeats occurred more than twice. The most frequent 99 bp repeat occurred 9 times. We obtained histograms of the long repeats by sorting the 99 bp sequences according to the starting position and counting the number of sequential starting positions.

Given the number of unique repeats with length L_test_ and the frequency of each repeat (frequency_repeat_), we calculated the probability that a DSB in a bacterial genome would lead to an incorrect final pairing. For each repeat, the number of incorrect targets in the genome is given by (frequency_repeat_ -1), whereas the number of correct targets is 1. Thus, the probability that a sequence matched pairing of the repeat is incorrect is (frequency_repeat_ -1)/ (frequency_repeat_). The probability that a DSB creates an invading strand terminating in this repeat is (frequency_repeat_)/(genome length). Thus, the total probability that an invading strand consisting of the particular repeat leads to an incorrect pairing is (frequency_repeat_ -1)/(genome length). Summing over the result for all unique repeats gives the total probability that a DSB will lead to a sequence matched incorrect pairing with length L_test_.

We note that all predictions of the exact probabilities for bacterial genomes are independent of the following: 1. The directionality of strand exchange. 2. Whether strand exchange is unidirectional or bidirectional. 3. Whether the bases are tested iteratively or all at once.

### Calculation of the number of distinct long repeat pairs in bacterial genome

To determine the prevalence of longer repeats, each possible 12 bp sequence was assigned a unique mapping number. The bacterial genome was divided into 12 bp sequences, each of which was assigned the corresponding mapping number. The 12 bp sequences were then sorted according to their mapping number, which grouped the entire genome into distinct 12 bp repeats. Longer repeats were probed by extending the 12 bp sequences within each mapping group and counting only those pairs that had no mismatches over the length L_test_. If the number is non-zero, that implies that there must be some repeats that have lengths ≥ L_test_. If most repeats only occur twice, the ratio of the number of distinct pairs to the length of the genome gives the probability that a DSB will lead to an incorrect pairing of that length.

### Probabilities of incorrect pairings for a bacterial genome using the simplified model with Chi sites that sparsely samples bacterial genomes

A DSB position in the genome was randomly chosen. The nearest Chi site on the 5′ side of the DSB was found. The 3′ end of the invading strand was positioned at the 3′ end of that Chi site. The homology test considers the L_test_ bases on the 5′ side of the Chi site.

### Simplified probabilistic model of homology testing in triplets

A random number generator creates a number between 0 and 1. Tripletpass_M_ describes the probability that a triplet with M mismatches will pass the triplet homology test. A triplet passes the homology test if Tripletpass_M_ is greater than the random number. We first considered homology tests with Tripletpass_M_ = 1 when M = 0, which means that the triplet is completely sequence matched. We considered two fundamentally different homology testing models. In one model, Tripletpass_M_ is the same for all M > 0. This represents the case in which collective interactions within the triplet allow a single mismatch to destabilize the triplet. For this model in which all triplets containing mismatches have the same probability of passing. We considered two different passing probabilities: Tripletpass_M_ = 0.25 and Tripletpass_M_ = 0.5.

We also considered models in which each mismatch decreases the probability that a triplet will pass the homology test. In that model, a random number generator creates a number between 0 and 4. We ran a model in which a homology test of a triplet passes the triplet if the number of mismatches is less than or equal to the value determined by the random number generator. Since 0 mismatches is less than or equal to all those values, a triplet with 0 mismatches has a 100% chance of passing the homology test. Similarly, 1 mismatch, 2 mismatches, or 3 mismatches have a 75%, 50%, or 25% chance of passing the homology test, respectively. We ran a second more promiscuous homology test in which 1 mismatch, 2 mismatches, or 3 mismatches have a 75%, 50%, or 50% chance of passing the homology test. Thus, the second test is the same as the first except for the probability that a completely mismatched triplet will pass the test.

## Results

### *In vitro* experiments probing RecA strand exchange in the presence of several periodically spaced single mismatches

Previous work has suggested that with ATP hydrolysis strand exchange can accept a single mismatch [17]. To gain some insight into the mismatch tolerance of triplet-based homology testing with ATP hydrolysis, we considered invading strands with periodically spaced single mismatches. How the invading strand is divided into triplet homology tests depends on the position at which strand exchange starts; however, periodically spaced single mismatches with 1 mismatch per 3 bases will always have exactly 1 mismatch/triplet. Similarly, periodically spaced single mismatches with 1 mismatch per 6 bases will have exactly 1 mismatch in every other triplet. The mismatch-containing triplets will be separated by single sequence-matched triplets.

**Fig 1:**
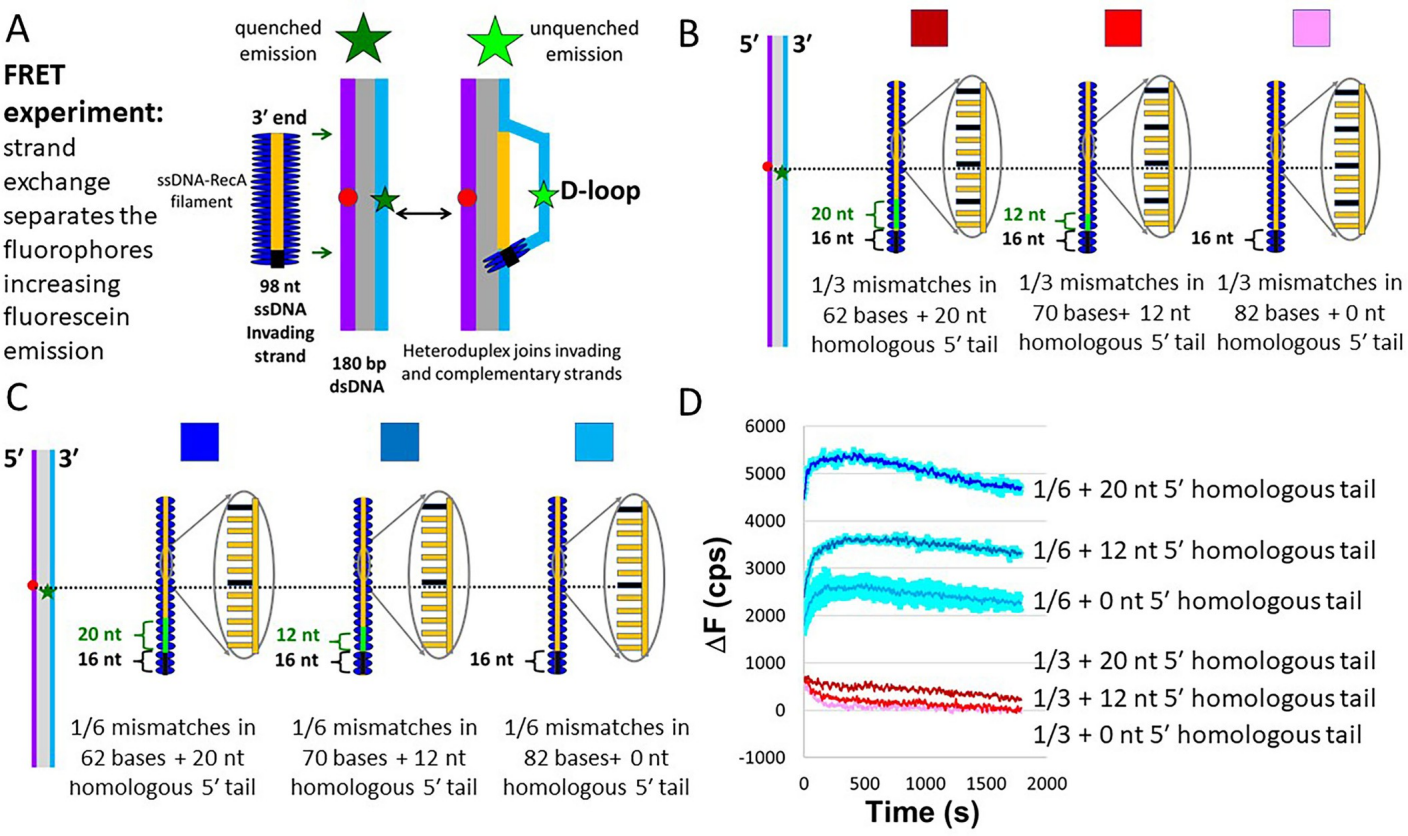
Strand exchange across single mismatches monitored using FRET. **(A).** Schematics of interactions of 180 bp dsDNA with 98-nt filaments containing different degrees of homology. The purple and light blue lines represent the complementary and outgoing strands, respectively. The gray rectangle indicates base paired regions. The dark blue ovals represent RecA. The red circle (rhodamine) and green star (fluorescein) show the locations of the fluorophores along the 180 bp dsDNA. All the invading strands contain 98 nt. The 16 heterologous bases nearest the 5′-end of the invading strand are shown in black. The green brackets indicate the region of the dsDNA that is partially homologous to the invading strand. The orange regions of the invading strand are partially homologous to the dsDNA. **(B).** Schematics of the experiments with mismatches periodically spaced 1 mismatch/3 bases. The orange regions of the invading strand include the periodically spaced mismatches, whereas the black and green regions are completely heterologous or completely homologous, respectively. The completely heterologous and homologous regions are also indicated by the black and green brackets. **(C).** Same as B but for invading strands with 1 mismatch every 6 bp. **(D).** Change in fluorescein emission (△F) vs. time curves. The dark red, bright red, and pink curves indicate results for 1/3 periodically spaced mismatches with a 20, 12, or 0 nt homologous tail on the 5′-side. The dark blue, medium blue, and light blue curves indicate results for 1/6 periodically spaced mismatches with a 20, 12, or 0 nt homologous tail on the 5′-side. Error bars represent root mean square deviation from two independent runs.

We observed strand exchange using FRET due to fluorophores positioned in the homoduplex (Fig 1A). When the homoduplex is base paired, emission of the fluorescein is quenched by the nearby rhodamine fluorophore. When base pairing is disrupted and both fluorophores are separated, fluorescein emission increases. In particular, strand exchange is probed by measuring the interaction between unlabeled 98-nt ssDNA/RecA filaments and labelled 180 bp dsDNA. The same dsDNA was used in all the experiments. The mismatches between the invading and complementary strands were controlled by the sequence of the invading strand (S1 Table).

The dsDNA had fluorophores positioned 90 bp (fluorescein, green star) and 89 bp (rhodamine, red circle) (Fig 1A) from the end of the dsDNA that is homologous to the 3′ end of the invading strand. The unlabeled ssDNA used for the fully homologous filament contained a 16-nt heterologous tail in order to have a total length of ∼ 100 bases (Fig 1A). The ∼ 100 nt length is long enough for ssDNA-RecA filaments to be stable with ATP hydrolysis. The ssDNA-RecA filament was mixed with the 180 bp dsDNA, and the resulting emission of the fluorescein label in the dsDNA was measured over time. To monitor strand exchange, we measure △F, the change in fluorescein emission, as a function of time.

In one set of experiments, the periodic mismatches were distributed over the entire invading strand (Fig 1B and 1C); therefore, strand exchange products with 1 mismatch/3 bases would be rejected by the initial 8 bp test [18], regardless of the tolerance of the triplet tests. To gain insight into the triplet tests we performed additional experiments in which the invading strands included homologous regions at the 5′ end that can pass the 8 bp test, as well as regions with periodically spaced mismatches that would be subject to triplet testing if the initial 8 bp test is passed (Fig 1B and 1C).

We considered interactions with 1 mismatch/3 bases (Fig 1B) and 1 mismatch/6 bases (Fig 1C). In the absence of a homologous 5′ tail, 1 mismatch/3 bases would always be rejected by the initial 8 bp test, and indeed we do not observe any significant fluorescence in that case (pink curve, Fig 1D). When a 12 or 20 nt homologous tail is present, 8 bp tests within the homologous tail can be passed. After that test is passed, strand exchange can progress through the remainder of the invading strand. Having a 12 nt homologous tail is insufficient to produce an increase in emission (Fig 1D, bright red curve) whereas a 20 nt tail might result in a very small increase in emission, but the increase is not statistically significant (dark red curve, Fig 1D). Thus, the results of invading strands with 1 mismatch/3 bases suggest that the triplet testing does not always pass triplets containing one mismatch.

Results of experiments with 1 mismatch/6 bases (Fig 1C) show that invading strands with 1 mismatch/6 bases can pass the initial 8 bp test (Fig 1D, blue curves). Thus, even without a homologous tail, invading strands with 1 mismatch/6 bases can provide information on the probability that a triplet with a single mismatch will be incorporated in a strand exchange product. Consistent with the results in yeast [12], interactions with 1 mismatch/6 bases show an increase in fluorescence due to formation of strand exchange products. The emission for 1 mismatch/6 bases was ∼ 25% of the emission for a perfectly matched invading strand. Thus, like Rad51 [12], RecA sometimes yields heteroduplex products from invading strands containing 1/6 = 16% mismatches. Unsurprisingly, the emission increases as the length of the homologous region at the 5′ end increases.

In these bulk experiments, we cannot determine the probability that a triplet containing a single mismatch will pass a homology test and be incorporated in a heteroduplex product; however, the probability that a triplet containing a single mismatch will pass a homology test is greater than 0, but less than 1.

### Analytical treatment of the deterministic homology testing L_test_ base pairs in a random genome

The *in vitro* experiments presented above suggest that RecA can form readily observable heteroduplex products that contain 16% mismatches. This poor homology stringency might indicate that RecA mediated homologous recombination alone could not lead to accurate DSB repair; however, statistical considerations show that highly mismatch tolerant homology testing of a large number of base pairs can provide extremely accurate homology recognition. Thus, we studied whether there are L_test_ values that could allow highly mismatch tolerant homology testing to almost always lead to correct DSB repair.

The *in vitro* results in this paper indicate the probability that a triplet containing a single mismatch will pass a homology test is greater than 0, but less than 1; however, we first consider simple deterministic homology testing models in which the probability of passing a test is always 1 for interactions that meet a homology threshold and always 0 for interactions that fail to meet that threshold. Such models can provide insight into how the accuracy of mismatch tolerant homology testing is influenced by L_test_, even if the models do not accurately capture the behavior of RecA mediated homologous recombination. Table 1 summarizes the terms used in the homology testing models.

**Table 1:** List of terms used in the homology testing models.

Term	Definition
Invading strand	ssDNA formed from one side of a DSB. RecA binds to the ssDNA, forming an ssDNA-RecA filament
Homoduplex DNA	dsDNA in the unbroken chromosome. It is composed of the complementary and outgoing strands.
Complementary strand	One of the DNA strands in the homoduplex. The ssDNA-RecA filament tests homology by trying to establish Watson-Crick pairing between the invading and complementary strands.
L_test_	Number of base pairs that are tested for homology to determine whether an attempted pairing between L_test_ bases in the invading and complementary strands should become permanent.
Heteroduplex product	dsDNA formed by Watson-Crick pairing of L_test_ bases in the invading and complementary strands after the homology test is passed. In the models, homology testing stops after the L_test_ bp heteroduplex product forms.
stringency	The fraction of interactions that forms an L_test_ bp heteroduplex product that correctly pairs a sequence region in the invading strand with the corresponding sequence region in the complementary strand.
α	When L_test_ bases are tested for homology as a group, the homology test is passed if the total number of mismatches in the L_test_ bases is ≤ ⟨ L_test_.
N_mismatch_	When deterministic homology testing is done in triplets, a triplet passes the homology test if the number of mismatches in the triplet is ≤ N_mismatch_.
N_mismatch8_	When deterministic homology testing includes an 8 bp test, the 8 bp test is passed if the number of mismatches in the 8 bp is ≤ N_mismatch8_.
Tripletpass_M_	When triplet homology testing is probabilistic, Tripletpass_M_ is the probability that a triplet will pass a homology test if the triplet contains M mismatches, where M = 0, 1, 2, or 3.

We begin by applying the deterministic models to “genomes” consisting of randomly chosen bases. We refer to these sequences as “random genomes”. We begin with random genomes for two reasons: 1. Simple analytical formulas describe the results of applying deterministic models to random genomes (Materials and Methods); and 2. Comparing results for random genomes to results for actual bacterial genomes allows us to highlight how the non-random nature of bacterial genome sequences could affect homologous recombination.

**Fig 2:**
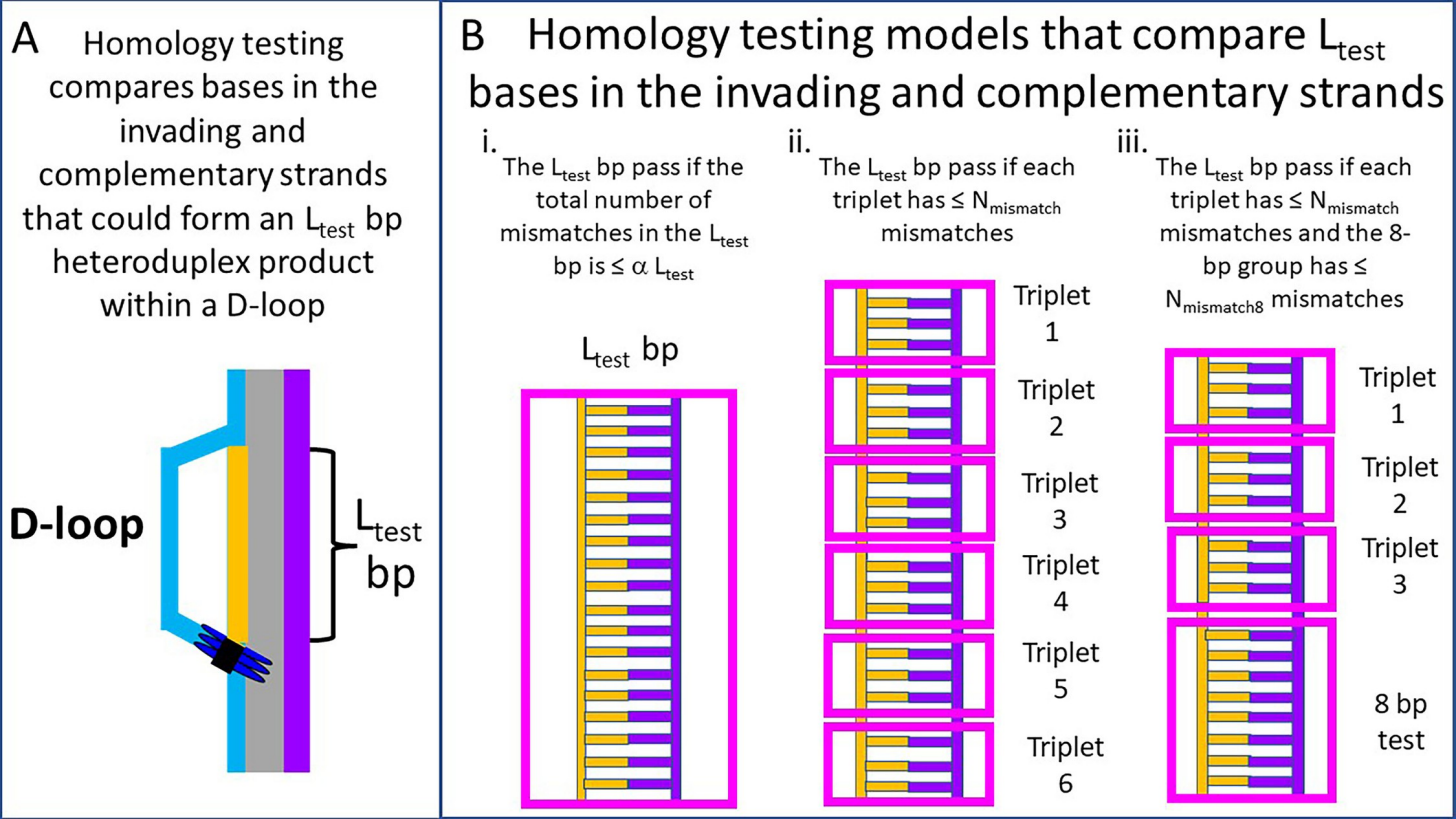
Illustrations of simple homology testing models that compare L_test_ base pairs in the invading and complementary strands. **(A)**. RecA mediated homologous recombination forms a D-loop in which the invading (orange) and complementary (purple) strands bind displacing the outgoing strand (blue). The gray regions indicate base pairing. **(B)**. Different homology testing models: i. the test is passed if the total number of mismatches in L_test_ bp is ≤ α L_test_. The strictness of the test depends on the value of α If α= 0, no mismatches are accepted. ii. The L_test_ base pairs are divided into triplets. Six separate triplets are shown. Each triplet is tested separately. An individual triplet passes the homology test if it includes ≤ N_mismatch_ mismatches. The L_test_ bp pass the homology test if every individual triplet passes the triplet test. If N_mismatch_ = 0, no mismatches are accepted. iii. Triplet testing is the same as in ii, but testing also includes an 8 bp test. The 8 bp test is passed if the 8 bp include ≤ N_mismatch8_ mismatches. The L_test_ bp pass the homology test if the 8 bp test is passed and every individual triplet passes the triplet test.

To highlight the influence of testing in triplets [22] and using an initial 8 bp test [16, 18–21], we consider three simple deterministic models (Fig 2). The first model considers the total number of mismatches in all L_test_ base pairs (Figs 2Bi, S1Ai, S1Bi and S1Ci). If the number of mismatches in L_test_ base pairs is > α L_test_, then the homology test always rejects the attempted base pairing and no stable heteroduplex product is formed. Otherwise, the homology test is passed, and a stable heteroduplex product is formed. Mismatch tolerance increases with α. Importantly, the result of the homology test is completely insensitive to the distribution of the mismatches within the L_test_ base pairs. We present this simple and very incorrect model to highlight how homology recognition is improved by dividing homology testing into base pair triplets and incorporating an initial 8-bp test.

**Fig 3:**
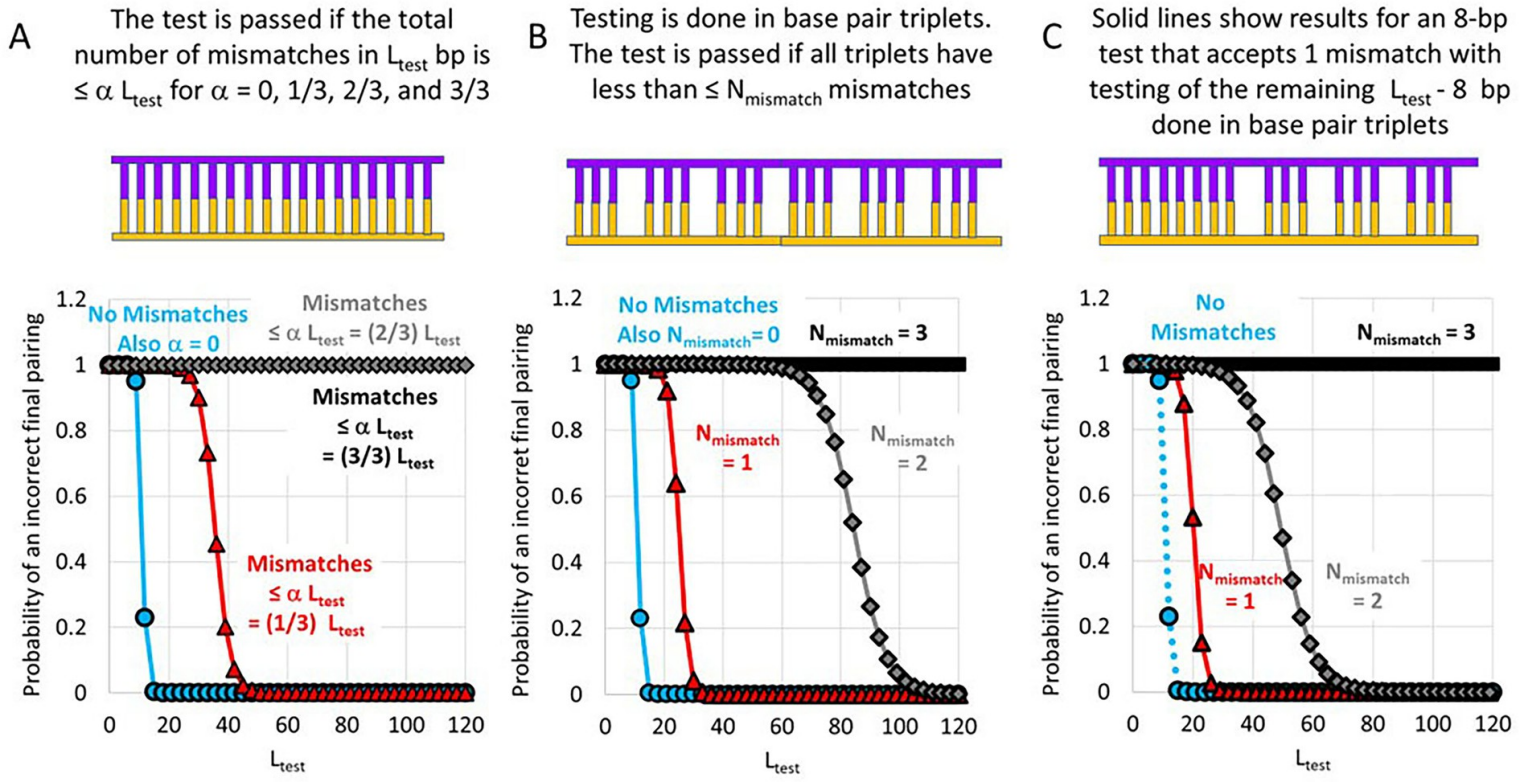
Probability of an incorrect final pairing vs. L_test_ for a random genome. In all panels, the light blue (circles) curve shows the result if no mismatches are accepted. **(A)**. The red (triangles), gray (diamonds), and black (squares) curves show the result if the number of accepted mismatches is αL_test_ = (m/3) L_test_ where m = 1, 2, or 3, respectively. The results for m = 2 and m = 3 are indistinguishable. (**B)**. The curves show the results in which the L_test_ base pairs are grouped into triplets. Each of the L_test_/3 triplets is tested for homology. The homology test of an individual triplet is passed if the number of mismatches in the triplet is less than N_mismatch_. All L_test_ base pairs pass the homology test if all L_test_/3 triplet homology tests are passed. The red, gray, and black curves with black outlined triangles, diamonds, and squares show the result for N_mismatch_ = 1, 2, or 3, respectively. (**C)**. The solid curves show results for homology testing that includes an 8-bp homology test that accepts one mismatch. (N_mismatch8_ = 1) and homology testing of (L_test_-8)/3 base pair triplets. The triangle, diamond, and square symbols indicate results for triplet tests that accept N_mismatch_ bp for N_mismatch_ = 1, 2, and 3, respectively.

The light blue lines with circular symbols in Fig 3 show the result for a homology test that does not accept any mismatches (α= 0). In the random genome, good stringency can be achieved by testing < 20 bp, which is why early work suggested that it was reasonable that RecA rejects homologous products that extend over less than 20 bp [11].

In Fig 3A, the curves indicated by the red triangles, gray diamonds, and black squares represent results for α= 1/3, 2/3, and 3/3, respectively. Fig 3A shows that homology testing of ∼ 50 bp provides good stringency for α= 1/3, but for α = 2/3, stringency is poor even if 120 bp are tested.

The second model divides the L_test_ base pairs into triplets, and then applies a homology test to each of the triplets (Figs 2Bii, S1Aii, S1Bii and S1Cii). Passing a homology test of L_test_ base pairs requires that no triplet includes more than N_mismatch_ mismatches. Otherwise, the homology test is always failed. The third model applies an 8 bp test that accepts one mismatch [18], with the remainder of the L_test_ -8 bases tested using the triplet test (S1Aiii, S1Biii and S1Ciii Figs). In both the second and third models, homology testing is sensitive to the distribution of mismatches within the L_test_ base pairs.

Fig 3B shows results for the model divides the L_test_ base pairs into triplets and then applies a homology test to each of the triplets. Again, there is a simple analytical formula that gives the stringency as a function of L_test_. In Fig 3B, the curves with the red triangles, gray diamonds, and black squares correspond to results for triplet tests with N_mismatch_ = 1, 2, and 3. Thus, the total number of allowed mismatches in Fig 3B is (N_mismatch_/3) L_test_. Importantly, for a given α, the total number of mismatches allowed in Fig 3A is α L_test_, and the symbolism for the curve in Fig 3A corresponding to α is the same as the symbolism in Fig 3B corresponding to N_mismatch_/3 = α. Thus, one can determine whether triplet testing improves stringency by comparing a curve in Fig 3B to the curve in Fig 3A that is represented by the same symbols.

The curves represented by the blue circles are identical in Fig 3A and 3B. Thus, if all mismatches are rejected, dividing homology testing into triplets offers no advantage. In contrast, if some mismatches are accepted, testing in triplets can bring a significant advantage. The advantage of triplet testing can be seen by comparing the curves represented by the red triangles and the gray diamonds. In Fig 3A, the curve with the gray diamonds indicates that α= 2/3 provides poor stringency even after testing 120 bp; however, Fig 3B indicates that testing in triplets allows N_mismatch_ = 2 to provide good stringency by testing only ∼ 50 bp.

Finally, we considered the third testing model based on previous work suggesting that in an initial 8-bp test [16, 18, 20, 21] is followed by testing in base triplets [22] (Fig 2Biii and S1Aiii, S1Biii and S1Ciii Fig). Again, there is an analytical formula for the stringency, and that formula is independent of the order in which the tests are performed. Thus, the results do not depend on the directionality of strand exchange or the order in which the 8-bp test and the triplet tests are performed.

The results for testing that includes an 8-bp test are shown by the solid lines in Fig 3C. Comparison between the solid curves with the same symbols in Fig 3B and 3C demonstrates that for both for N_mismatch_ = 1 (red triangles) and for N_mismatch_ = 2 (gray diamonds), the initial 8-bp test decreases the L_test_ required to achieve a given stringency. The required L_test_ decreases because the 8 bp test is stricter than either triplet test. Interestingly, with an 8 bp test, even triplet tests that accept 2 mismatches can provide ∼99% stringency by testing ∼ 75 bp. For N_mismatch_ = 3 (black squares) the 8 bp test offers a negligible improvement because the triplet test never rejects any pairing. In sum, Fig 3 shows that dividing L_test_ into groups that are tested for homology separately can greatly improve the stringency of mismatch-tolerant homology testing; however, the stringency of mismatch intolerant testing is not affected by dividing L_test_ base pairs into groups. We note that all the results in Fig 3 are independent on whether homology testing occurs iteratively or simultaneously (Fig 3); however, iterative testing vastly improves searching speed. Furthermore, in iterative testing, searching speed improves if the 8 bp test occurs before the triplet tests.

Importantly, though different curves in Fig 3 represent various homology testing models, all the curves show that stringency as a function of L_test_ divides into two regimes. At low values of L_test_, most sequences can pass a homology test at several positions in the genome; therefore, stringency is very poor and insensitive to L_test_. At higher values of L_test_, most sequences can only pass the homology test at the corresponding position in the genome, and stringency increases exponentially with L_test_. Of course, the L_test_ value that divides the regime increases as homology testing becomes less strict.

### Deterministic homology testing in bacterial genomes

Bacterial genomes do not consist of randomly chosen bases. Instead, they contain long repeats. S2A Fig shows histograms of the distribution of repeats in *E*.*coli* MG1655 that are longer than 99 bp. Importantly, there are 900 positions in a 1000-bp repeat at which a DSB could create an invading strand that includes 100 bp that occur at least one other position in the genome. Thus, S2B Fig shows a graph of the number of times that unique 100-bp sequences appear in the genome.

To determine the stringency for a homology test that considers L_test_ base pairs in the *E*. *coli* MG1655 genome and rejects all attempts to form a heteroduplex product that includes mismatches, we counted the number of 100-bp sequences that occur more than once in the given strand (Materials and Methods). Even a homology test that rejects all mismatches could not reject sequences that join different copies of these 100-bp repeats. S2C Fig indicates that there are more than 13,000 unique 100-bp sequences that occur exactly twice in the given strand of the *E*. *coli* MG1655, and one 100-bp sequence has 9 copies in that given strand.

**Fig 4:**
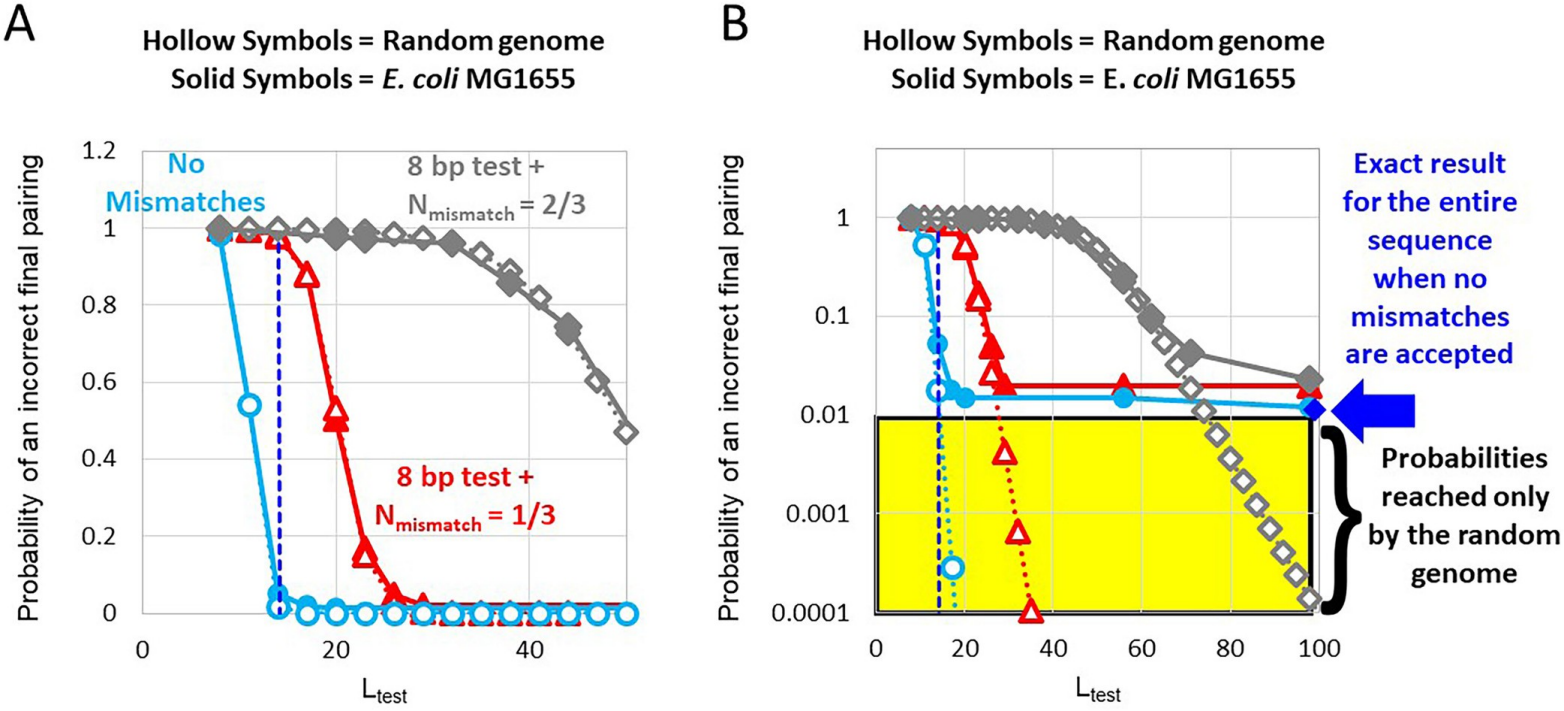
Probability that after a DSB RecA will create a final incorrect pairing as a function of L_test_ for the random genome (hollow symbols) and the *E*. *coli* MG1655 genome (solid symbols). (**A)**. The probability that a final pairing will be incorrect as a function of L_test_ and N_mismatch_. The blue line shows results for sparse homology testing that requires complete sequence matching. The red and gray lines with triangle and diamond symbols indicate results for sparse homology testing when the 8 bp test that accepts one mismatch is followed by triplet tests that accept 1and 2 mismatches, respectively. The dark blue vertical dashed line indicates L_test_ = 14. (**B)**. Same as A with a logarithmic y-axis. The blue diamond highlighted by the blue arrow indicates the exact result for homology testing the entire genome when L_test_ = 98 and no mismatches are accepted. The yellow region indicates the stringencies that are achieved in the random genome but are not reached in the *E*. *coli* MG1655 genome.

For this work, we make the simplifying assumption that DSB are uniformly distributed across the genome. Furthermore, we assume that homology testing is also uniformly distributed across the genome. Given those assumptions, there is a >1% probability that a DSB at a random position in the *E*. *coli* MG1655 genome would lead to a sequence matched heteroduplex product that joins two different copies of a 100 bp sequence. The dark blue diamond in Fig 4B indicates the results of this calculation.

We now compare the results for the random genomes to the results for *E*. *coli* MG1655 (Fig 4A and 4B). The light blue lines in Fig 4B indicate that when L_test_ = 21, if no mismatches are accepted, the stringency for random genomes is ∼ 1-1x10^-6^, which is much better than 99%. The stringency in the random genome is high because in a random genome the probability that two bases accidentally match is ¼ (S3 Fig). Thus, the probability that two 21-bp sequences in a random genome match is 1/4^21^∼2x10^-13^. This low probability implies that random genomes do not contain long repeats. Additionally, the probability that a 21-bp sequence has a match in a 5x10^6^ nt sequence is 2x10^-13^ x 5x10^6^ ∼ 10^−6^, consistent with the result shown by the dotted light blue line in Fig 4B. Therefore, Fig 4B indicates that if no mismatches are accepted the stringency achieved in a random genome when L_test_ = 21 is much better than the stringency achieved in a bacterial genome when L_test_ = 100 bp because when L_test_ > 20 bp most incorrect pairings in bacterial genomes join different copies of repeats. In sum, the long repeats in bacterial genomes limit stringency to ∼ 99% unless L_test_ is larger than 100 bp.

It is worth noting that stringency for bacterial genomes stringency cannot be expressed by a simple analytical formula, and the search technique that we used to find the exact matches does not extend to inexact matches. Thus, we used computer simulations to study how mismatch tolerance influences stringency in bacterial genomes (Materials and Methods). The solid curves with circular symbols in Fig 4 show results for computer simulations of homology testing using an 8-bp test that accepts one mismatch and triplet testing of L_test_-8 base pairs for an *E*. *coli* MG1655 genome. For comparison, results for this test in a random genome are shown by dotted curves with hollow-square symbols. In both cases, the blue curves show results for tests that reject all mismatches. The remaining curves show results for an 8-bp test that accepts one mismatch, where the remainder of the L_test_-8 bases are then tested in base pair triplets. The red and gray curves show results for triplet tests that accept 1 and 2 mismatches, respectively. The results for the random genome and the bacterial genome seem nearly identical (Fig 4A). Fig 4B shows the same results as Fig 4A, except that the y axis is logarithmic. In Fig 4B, it is clear that the results for the random genome and the results for the bacterial genome diverge in the region where stringency > 99% because at those stringencies most incorrect heteroduplex products join different copies of long repeats.

For L_test_ values that give poor stringency in the random genome, the *E*. *coli* results agree well with the results for the random genome (Fig 4) because most groups of L_test_ bp that pass the homology test in the *E*. *coli* genome do not join different copies of long repeats. In contrast, for L_test_ values that provide > 99% stringency in a random genome (yellow highlighted region in Fig 4B), the *E*. *coli* results show much poorer stringency than the random genome. Importantly, once the *E*. *coli* stringency reaches ∼ 99%, increasing L_test_ does not improve stringency. This saturation of stringency occurs for both sparse sampling and complete sampling (S4 Fig) because most incorrect groups of L_test_ base pairs that pass the homology test join different copies of long repeats (S5 Fig). Other bacterial genomes also show a similar saturation of stringency with L_test_ because they too contain long repeats (S6–S8 Figs). In sum, independent of any feature of RecA-mediated homologous recombination, if the 3′ end of the invading strand is located at a random position in the genome, then even if homology testing rejects all mismatches more than 1% of the 100 bp groups that pass the homology test would include complementary and invading strands from different copies of long repeats (Fig 4). Furthermore, if testing rejects all mismatches, then testing more than ∼ 14 bp does not significantly improve stringency.

After a DSB, if repair follows the RecBCD pathway the RecBCD protein interacts with the ends of the broken dsDNA [2, 26–28]. The function of RecBCD changes when it recognizes the 8-bp sequence GGCGGCGG, which is called the Chi site [28–30]. If there were no Chi sites in long repeats, then no heteroduplex product could join different copies of long repeats if the RecBCD pathway were followed; however, some Chi sites are positioned in long repeats [31]. Thus, to determine how terminating invading strands at or near Chi sites influences mismatch-tolerant homology recognition, we performed various simulations.

For Chi site simulations, the DSB occurs at a randomly chosen position in an *E*. *coli* MG1655 genome; however, the invading strand sequence used in the simulation terminates in the nearest Chi site on the 5′ side of the DSB. The homology test is then applied to the L_test_ base pairs on the 5′ side of the Chi site. We note that regardless of the direction of strand exchange, these are the L_test_ base pairs that are included in the heteroduplex that extends to the 3′ end of the invading strand.

There are ∼ 500 Chi sites in each strand of the 4.6 Mbp *E*. *coli* MG1655 genome [26]. Thus, the separation between Chi sites frequently extends over thousands of base pairs. As a result, thousands of different DSB positions will produce the same invading strand sequence. A detailed consideration of the distribution of Chi sites in genomes is required to determine whether the RecBCD pathway increases or decreases the probability that one side of a DSB will lead to a pairing between different copies of a long repeat. Interestingly, our simulations indicate that stringency as a function of L_test_ is not affected by whether or not the invading strand terminates in a Chi site (S8 Fig). These simulations only consider the invading strand formed by one side of the DSB. If the RecBCD pathway is followed, no single DSB could create two invading strands from the same long repeat [31].

Importantly, though different curves in Figs 4 and S6 represent various homology testing models, all the curves show that stringency as a function of L_test_ divides into three regimes. The first two regimes are shared with random genomes; however, for bacterial genomes an additional new regime begins when stringency as a function of L_test_ saturates. That asymptotic value occurs because pairings join different copies of long repeats. Finally, for bacterial genomes there is an eventual increase in stringency once L_test_ ∼ 1000 bp (S4 Fig), consistent with the histogram of repeat lengths shown in S2 Fig.

### More realistic homology testing

The results of the deterministic homology recognition models provide insight into how L_test_ affects stringency. The results also highlight how long repeats in bacterial genomes limit the accuracy of homology testing, even if the testing does not accept any mismatches. Unfortunately, our *in vitro* results indicate that the deterministic models do not accurately capture homology testing by RecA; the *in vitro* results suggest that 1-bp mismatch is sometimes accepted and that 1-bp mismatch is sometimes rejected, whereas in the deterministic model the mismatch would either always be accepted or always be rejected. Therefore, we also considered models where the acceptance of mismatches is probabilistic. For example, in such models the probability of accepting a single mismatch in a triplet might be 75%, which implies that the probability that the mismatch would be rejected would be 25%.

Unfortunately, the bulk FRET measurements in Fig 1 do not allow us to accurately determine the probability that a triplet homology test will accept 1, 2, or 3 mismatches within that triplet. Thus, we considered various probabilistic models. In the models, the probability of passing a triplet test is characterized by tripletpass_M_, where M represents the number of mismatches in the triplet. It is known that RecA family proteins sometimes reject perfectly homologous sequences [32]. Thus, one might think that it would be useful to run simulations in which perfectly homologous triplets are sometimes rejected (tripletpass_0_ < 100%); however, if for all M tripletpassM is proportional to tripletpass_0_, then the stringency vs L_test_ for a homology test with tripletpass_0_ < 100% is same as the stringency vs L_test_ for tripletpass_0_ = 100%. Reducing tripletpass_0_ below 100% increases searching times; therefore, for all the results shown in Fig 4, we chose tripletpass_M_ = 100% for completely matched triplets. This is perfectly compatible with many sequence-matched products reversing before they extend over L_test_ base pairs. Indeed, simulations in which perfectly matched sequences could reverse before reaching L_test_ showed the same stringency vs. L_test_ as simulations in which sequence-matched regions never reverse.

The various models we considered may not exactly capture the details of RecA-mediated homologous recombination *in vivo*, but they allow us to test whether stringency as a function of L_test_ is very sensitive to the details of the probabilistic homology testing. If the results of our models are insensitive to details of the models, then the modeling results may provide insight to mismatch tolerant homology testing by RecA.

**Fig 5:**
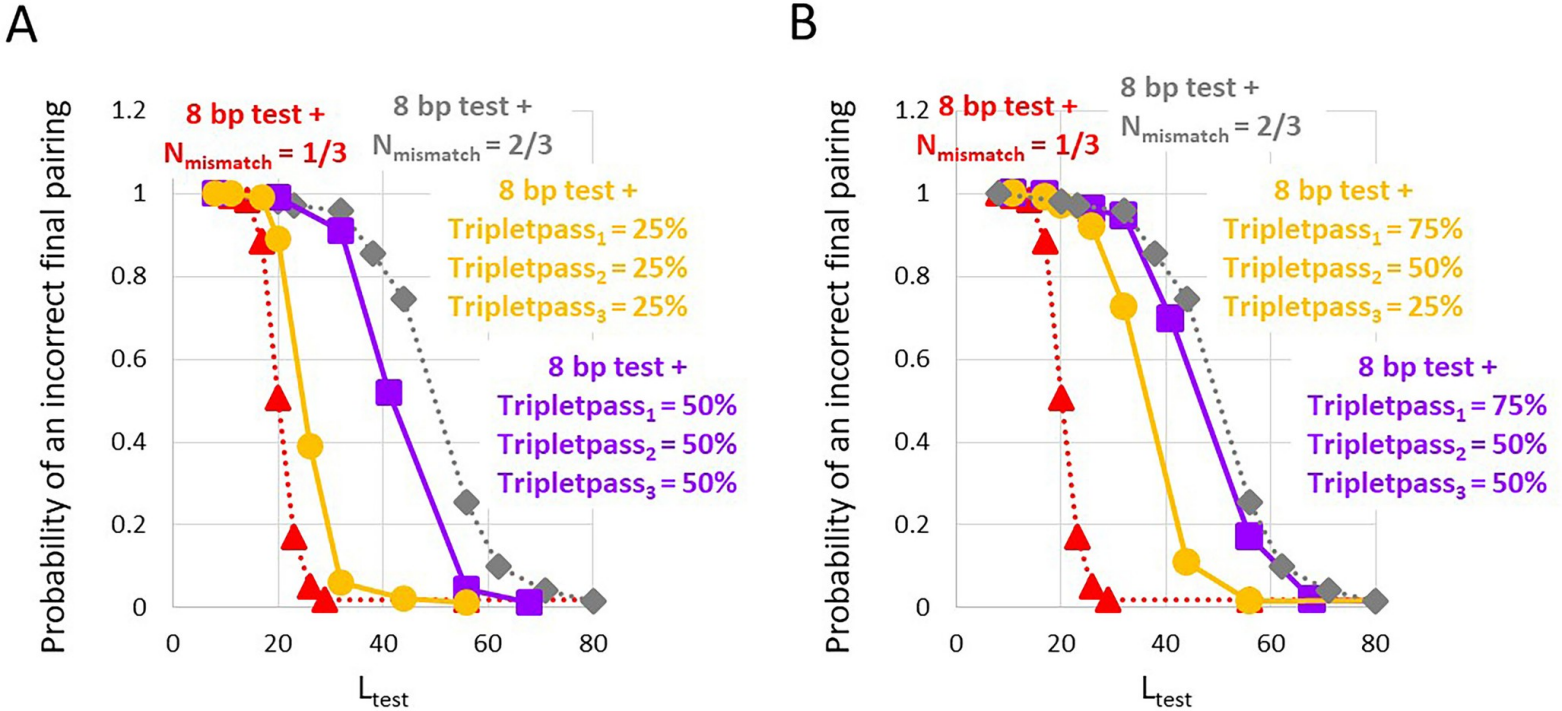
Probability that after a DSB RecA will create a final correct or incorrect pairing as a function of L_test_ for *E*. *coli* MG1655 genome for deterministic and probabilistic homology testing. (**A)**. Probability that a final pairing will be incorrect as a function of L_test_ and N_mismatch_. The red and gray lines with triangle and diamond symbols indicate results for sparse homology testing when the 8-bp test that accepts one mismatch is followed by triplet tests that accept 1 or 2 mismatches, respectively. The orange and purple curves represent various probabilistic testing strategies. For both the orange and purple curves tripletpass_M_ = 100% for completely matched triplets. The orange and purple curves with the circular and square symbols represent results for homology tests in which tripletpass_M_ = 25% or 50%, respectively, for any triplet that contains at least one mismatch. **(B)** The curves with the triangular and diamond symbols are the same as in A. For both the orange and purple curves tripletpass_M_ = 100% for completely matched triplets. The orange curve shows results for tripletpass_M_ = 75%, 50%, and 25% for 1, 2, and 3 mismatches, respectively. The purple curve shows results when tripletpass_M_ = 75%, 50%, and 50% for 1, 2, and 3 mismatches, respectively.

Results of probabilistic homology testing are shown in Fig 5. The triangle and diamond symbols in Fig 5 repeat the curves shown in Fig 4 that indicate results of deterministic homology testing of *E*. *coli* MG1655 genomes using an initial 8-bp test followed by triplet tests that reject any triplet with more than N_mismatch_ mismatches. The results of the deterministic tests can be compared with results of several different probabilistic testing models that include an initial 8-bp test that is followed by triplet tests that depend on the number of mismatches in each triplet.

Previous work has indicated that collective interactions between bases might allow a single mismatch to destabilize a triplet [22, 33, 34]. In that case, tripletpass_M_ would be the same for all M > 0. In Fig 5A the orange and purple curves show results for tripletpass_M_ = 25% or 50%, respectively, for all M > 0. For the data shown in Fig 1, when there is a 20-nt homologous tail the invading strand with 1 mismatch per 6 bases includes 10 mismatched triplets. Thus, the probability of getting through all 10 mismatches would be .25^10^<10^−6^ or .5^10^<10^−3^. Of course, a FRET signal may not require progressing through all the mismatched triplets.

To further probe the robustness of the results, we considered probabilistic models in which each additional mismatch decreases the stability of the triplet. In this case tripletpass_M_ would decrease with M (orange and purple curves in Fig 5B). The orange curve shows results for tripletpass_M_ = 75%, 50%, and 25% for M = 1, 2, and 3 mismatches, respectively; therefore, for tripletpass_M_ = 75% the probability of getting through all 10 mismatched triplets would be .75^10^ ∼ 6%.

For bacterial genomes the predicted stringencies as a function of L_test_ fall in the range between the predictions of the simplistic deterministic model for N_mismatch_ = 1 (Fig 5, red curve) and N_mismatch_ = 2 (Fig 5, gray curve). We also considered other probability distributions that are compatible with the *in vitro* results shown in Fig 1, and those results also fall largely in the same range. In sum, for probabilistic models consistent with the *in vitro* results shown in Fig 1, the stringencies as a function of L_test_ are insensitive to the details of the models, including whether or not all mismatched triples have the same probability.

## Discussion

Many aspects of RecA homology testing are not fully understood. In agreement with previous published results [13–17], this work presented *in vitro* data suggesting that even with ATP hydrolysis, RecA homology testing is highly mismatch tolerant (Fig 1). We now consider origins and implications of the high mismatch tolerance of RecA mediated homologous recombination.

Searching speed is an important requirement for many sequence testing systems. We speculate that highly accurate sequence recognition requires rigidly held Watson-Crick partners that would slow homology testing; therefore, we propose that the mismatch tolerance of RecA is a consequence of structural features that speed DSB repair.

Importantly, the modeling results in this paper suggest that for bacterial genomes stringency vs. L_test_ for bacterial genomes divides into three regimes: 1. At small L_test_, stringency is very poor and insensitive to L_test_ because almost every possible sequence of length L_test_ occurs many times in the genome. 2. At moderate L_test_, stringency increases exponentially with L_test_ because most sequences of length L_test_ occur only once in the genome. 3. At large L_test_, stringency saturates because testing more bases does not improve stringency since most incorrect products join different copies of long repeats.

We now review results of recombination frequency as a function of the homologous sequence length, L. Significant recombination requires a homologous segment that extends over more than 20 bp [9–11]. The frequency of recombination increases approximately exponentially as L increases from ∼20 to ∼75 bp; however, when L is larger than approximately 75 bp, recombination increases linearly with L [10]. Thus, the recombination frequency as a function of L divides into three distinct regimes: 1. For L < 20 bp recombination is negligible. 2. For 20 < L < 75 bp recombination shows a steep exponential increase with L. 3. For L > 75 bp there is a very slow linear increase in recombination with L. In sum, both recombination as a function of L and predicted stringency as a function of L_test_ divide into three regimes.

We propose that highly mismatch tolerant homology testing underlies the following L dependent features of recombination: 1. Recombination is negligible when L < 20 because when L_test_ < 20 bp almost all products are wrong, whereas almost all 20 bp products are correct if testing rejects all mismatches. 2. Recombination increases strongly with L for 20 ≤ L ≤ 75 bp because stringency increases strongly with L_test_ from 20 ≤ L ≤ 75 bp. 3. Recombination increases slowly with L when L > 75 bp because testing more bases does not significantly improve stringency since most incorrect products join different copies of long repeats (Figs 3–5).

Finally, this work has shown that the stringency that can be achieved by RecA alone depends strongly on L_test_, so it is important to consider what might govern L_test_ *in vivo*. With ATP hydrolysis even 150 bp products are highly unstable [25]. In contrast, *in vivo* incorporation of regions of accidental homology saturates at ∼ 75 bp [9, 10]; therefore, *in vivo* L_test_ is unlikely to be governed by the length-dependent stability of heteroduplex products. Thus, we speculate that irreversible alignment between the broken and unbroken chromosomes usually depends on significant polymerization by DNA polymerase Pol IV [35, 36], which requires that heteroduplex products extend over ∼ 50–75 bp [37].

Other proteins may also influence the stringency of DSB repair. Even after testing 98 bp, some products of highly mismatch tolerant testing could contain mismatches (S9 Fig). MutS, MutL, and UVrD could combine to reverse sufficiently mismatched heteroduplex products [38], consistent with recombination of closely related bacterial genomes being blocked by MutS [39].

Finally, the ∼1% of DSB repairs that join different copies of repeats would allow extensive Pol IV polymerization and would not be reversed by mismatch repair. Thus, if those pairings are not reversed by another protein, the pairings could lead to cell death or to genomic rearrangement.

In sum, we propose that the level of genomic alterations produced during recombinational repair reflects a critical balance between highly mismatch-tolerant RecA-mediated strand exchange and intervention by other cellular components. The balance is presumably tuned by evolutionary forces to meet the requirements of rapid repair, genetic stability, and genetic variation, which may vary according to the cellular environment.

## Supporting information

S1 Fig Illustration of different homology testing schemes. (DOCX)

S2 Fig Distributions of repeated sequences in the *E*. *coli* MG1655 genome. (DOCX)

S3 Fig Enumeration of all 16 possible base pair sequences. (DOCX)

S4 Fig Predicted incorrect DSB repairs as a function of L_test_ for N_mismatch_ = 0 for sparse sampling and complete sampling. (DOCX)

S5 Fig Probability that a DSB will result in an incorrect final pairing vs. L_test_ if all mismatches are rejected for different bacterial genomes. (DOCX)

S6 Fig Saturation in the decrease in incorrect pairings as L_test_ increases. (DOCX)

S7 Fig Ratio of the number of distinct pairs of starting locations in the given strands of bacterial genomes that share a repeat of length L to the genome length as a function of L. (DOCX)

S8 Fig Probability that a DSB will result in an incorrect final pairing vs. L_test_ if invading strands all terminate in Chi sites. (DOCX)

S9 Fig Predicted incorrect DSB repairs for L_test_ = 98. (DOCX)

S1 Table Sequences used for experiments in Fig 1. (TIF)
