# Supplementary material for: Highly mismatch-tolerant homology testing by RecA could explain how homology length affects recombination
Source: PLoS One. 2023 Jul 13;18(7):e0288611. doi: 10.1371/journal.pone.0288611 (PMC10343044; doi:10.1371/journal.pone.0288611)
Supplement: S1 Fig — (DOCX) [file pone.0288611.s001.docx]

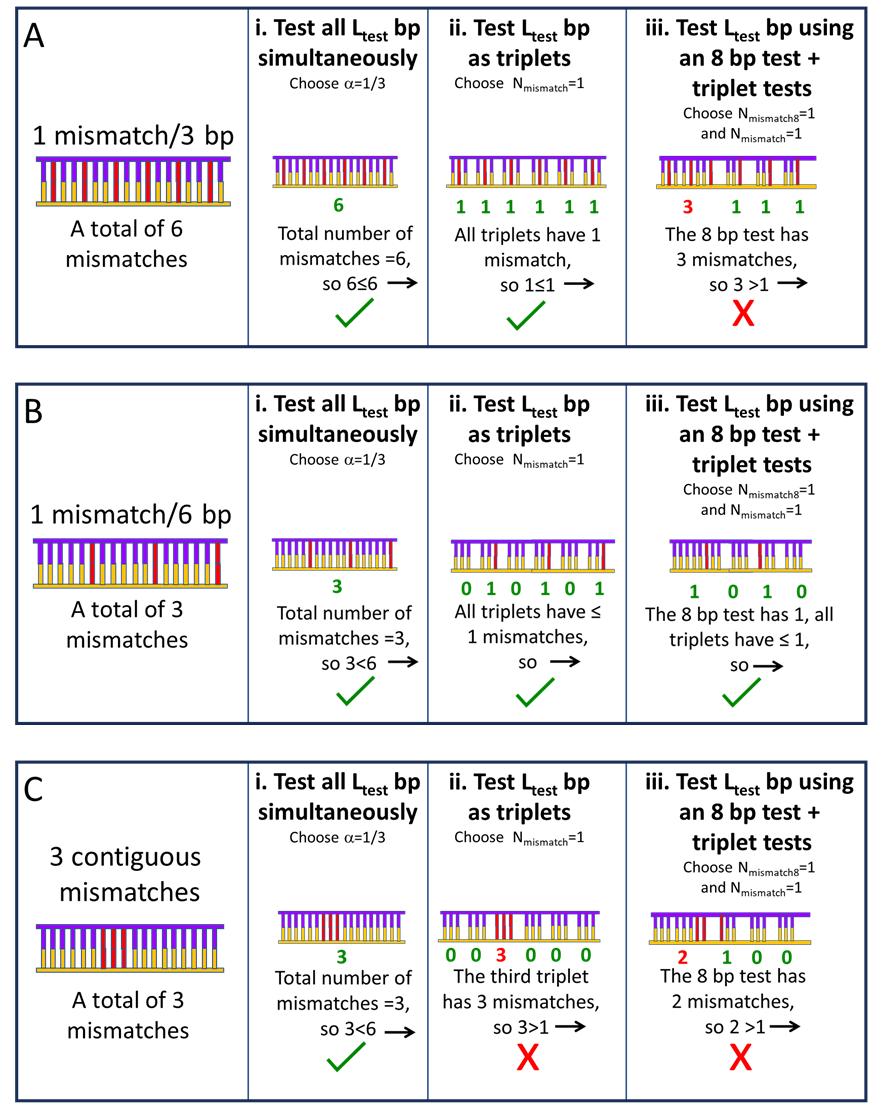


**S1 Fig. Illustration of the homology testing outcomes for 3 different sequences and the three testing strategies illustrated in Fig 2.** Green checks indicate passes, red x’s indicate fails. For each test, the bases are divided into the test groups, and the number of mismatches/group are indicated below each group. Green and red numbers indicate passes and fails, respectively. **(A).** A sequence with periodically spaced mismatches 1/3 bp. (i). When α = 1/3, the sequence will pass all homology tests that consider all L_test_ bases at once. (ii). When N_mismatch_ = 1, the sequence will also pass all homology test that groups the bases into triplets. (iii). When N_mismatch8_ = 1, the sequence always fails any 8 bp test. **(B).** A sequence with periodically spaced mismatches 1/6 bp. (i). When α = 1/3, the sequence will pass all homology tests that consider all L_test_ bases at once. (ii). When N_mismatch_ = 1, the sequence will also pass all homology tests that group the bases into triplets. When N_mismatch8_ = 1, the sequences will pass some 8 bp tests (iii). The same sequence will fail 8 bp tests that include 2 mismatches, which can occur if a mismatch occupies the first or second position in the 8 bp groups. **(C).** A sequence with one group of 3 contiguous mismatched triplets. (i). When α = 1/3, the sequence will pass all homology tests that consider all L_test_ bases at once if L_test_ ≥ 9 bp. (ii). When N_mismatch_ = 1, the sequence will fail one triplet test. (iii). When N_mismatch8_ = 1, the sequences will pass fail the 8 bp test.
