# Supplementary material for: Highly mismatch-tolerant homology testing by RecA could explain how homology length affects recombination
Source: PLoS One. 2023 Jul 13;18(7):e0288611. doi: 10.1371/journal.pone.0288611 (PMC10343044; doi:10.1371/journal.pone.0288611)
Supplement: S2 Fig — (DOCX) [file pone.0288611.s002.docx]

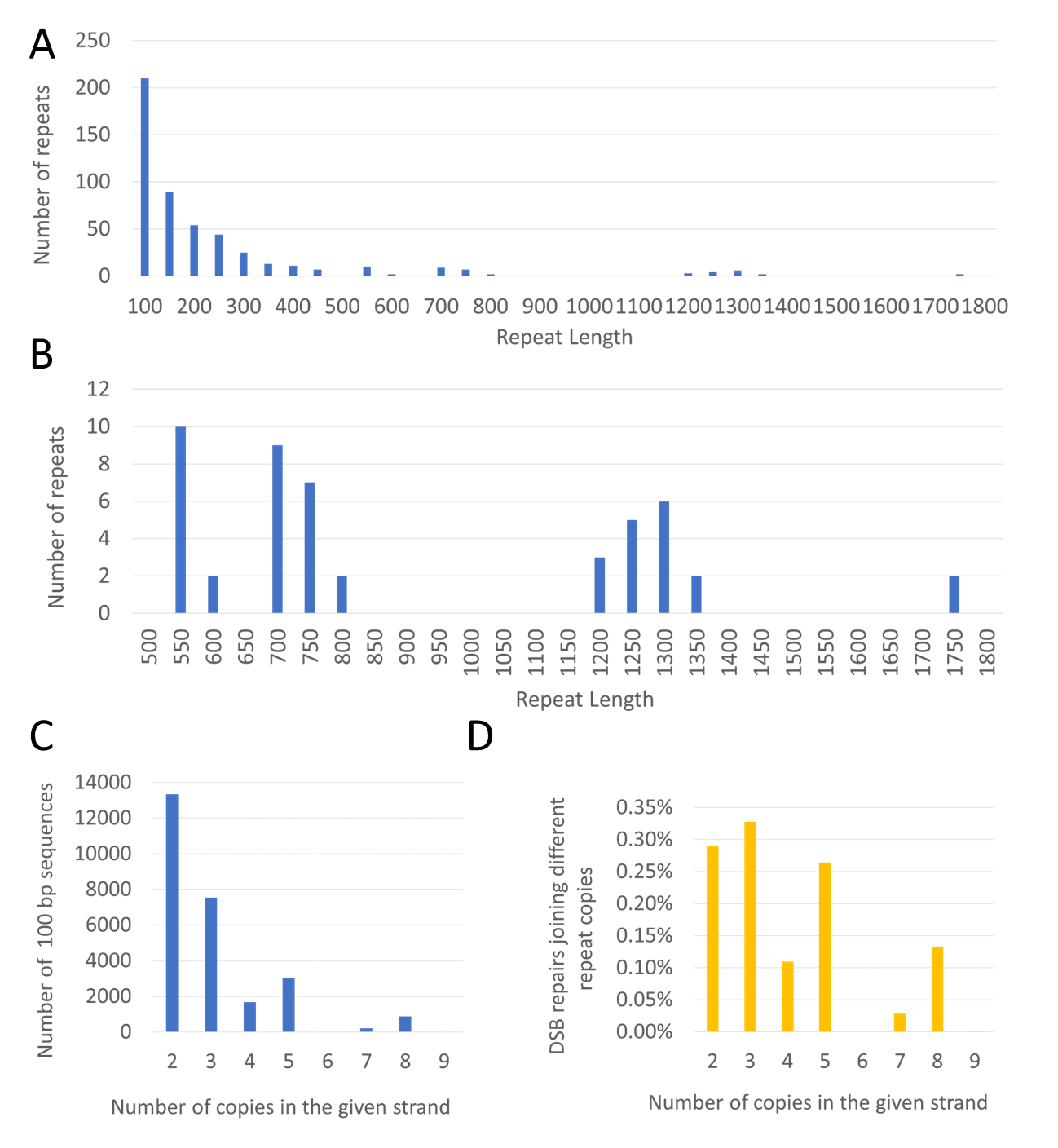


**S2 Fig.** **Distributions of repeated sequences in the *E. coli* MG1655 genome. (A).** Histogram of the distribution of repeat lengths in *E. coli* MG1655. **(B).** Same as A but only showing repeats longer than 500 nt. **(C)**. Graph of the number of unique 100-bp repeats in the *E. coli* MG1655 genome as a function of the number of times the 100-bp sequence is repeated. More than 13000 different sequences are repeated exactly twice, whereas one sequence is repeated 9 times. **(D)**. Graph of the probability that a DSB at a random position in the genome would lead to a pairing between different copies of 100-nt sequences as a function of the frequency of the repeat. More frequent sequences have a higher probability because each invading strand has more possible incorrect targets.
