# Supplementary material for: Highly mismatch-tolerant homology testing by RecA could explain how homology length affects recombination
Source: PLoS One. 2023 Jul 13;18(7):e0288611. doi: 10.1371/journal.pone.0288611 (PMC10343044; doi:10.1371/journal.pone.0288611)
Supplement: S3 Fig — (DOCX) [file pone.0288611.s003.docx]

**S3 Fig. List of all 16 possible base pair sequences.** The first column enumerates the base pairs. The second column lists each of the four possible first bases. Each base is repeated 4 times. The second column lists all 4 possible partners for each of the four bases. The third column illustrates the 4 correct pairings out of the 16 possible base pair sequences.
