# Supplementary material for: Highly mismatch-tolerant homology testing by RecA could explain how homology length affects recombination
Source: PLoS One. 2023 Jul 13;18(7):e0288611. doi: 10.1371/journal.pone.0288611 (PMC10343044; doi:10.1371/journal.pone.0288611)
Supplement: S4 Fig — (DOCX) [file pone.0288611.s004.docx]

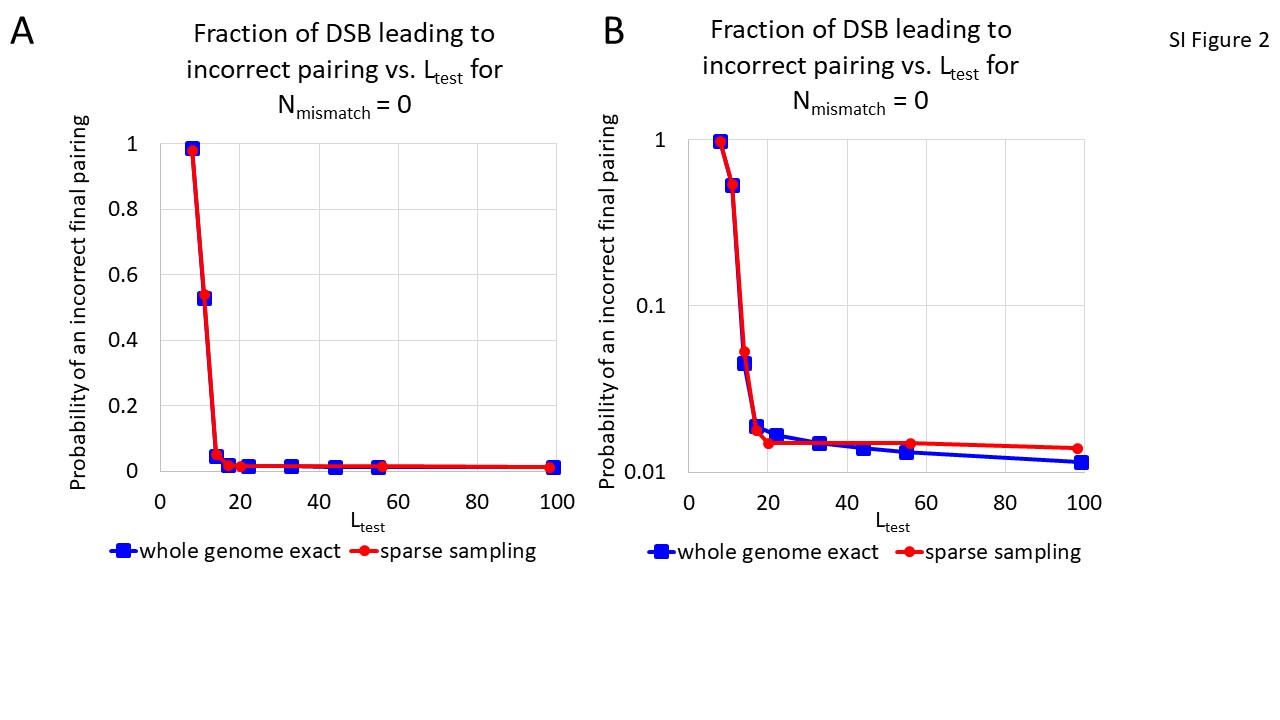


**S4 Fig. Predicted incorrect DSB repairs as a function of L_test_ for N_mismatch_ = 0 for sparse sampling and complete sampling.** **(A).** The red line with circular markers is the same as the red line in Fig1, which is the result for sparse sampling. The blue line with square markers is the result for complete sampling of the *E.coli* MG1655 genome. Results are very similar. The error in the sparse sampling masks the small increase in stringency that occurs as L_test_ is increased from 17 to 99. **(B).** Same as A but with a logarithmic y axis.
