# Supplementary material for: Highly mismatch-tolerant homology testing by RecA could explain how homology length affects recombination
Source: PLoS One. 2023 Jul 13;18(7):e0288611. doi: 10.1371/journal.pone.0288611 (PMC10343044; doi:10.1371/journal.pone.0288611)
Supplement: S5 Fig — (DOCX) [file pone.0288611.s005.docx]

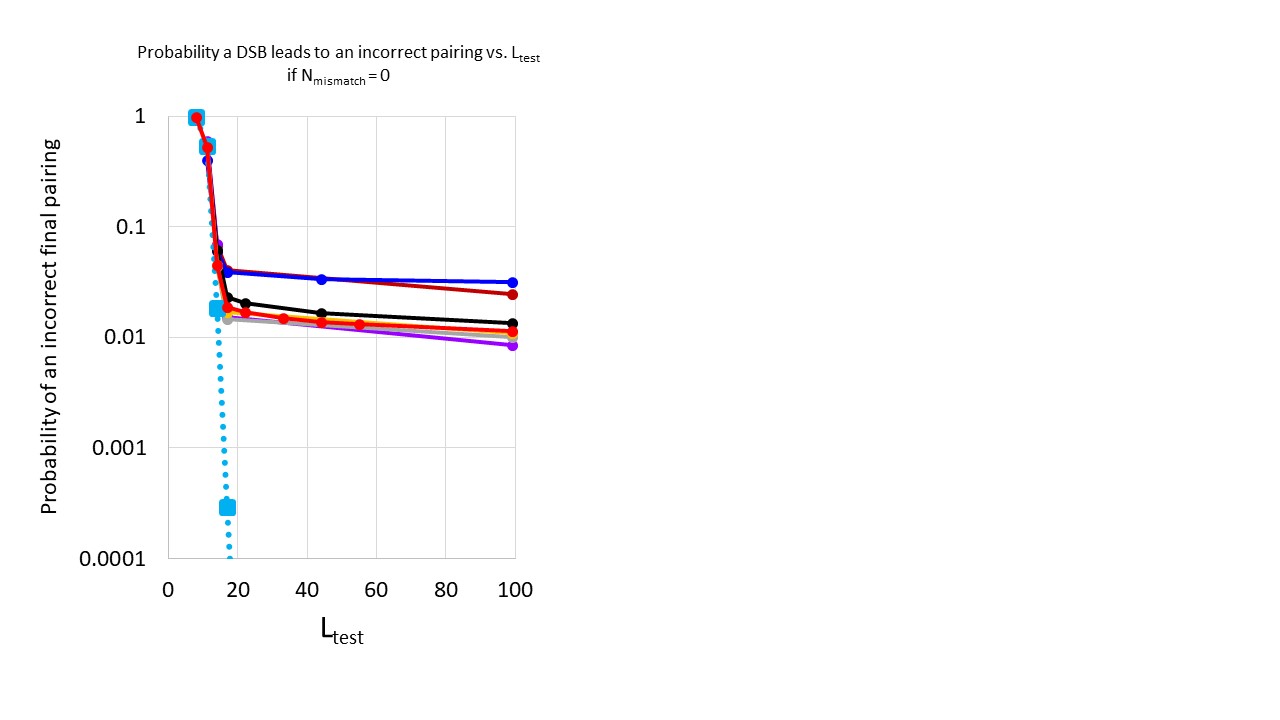


**S5 Fig.** **Probability that a DSB will result in an incorrect final pairing vs. L_test_ if all mismatches are rejected for different bacterial genomes.** The dotted blue line represents results for a random genome and are the same as those shown in Fig 3. The colors of the other curves correspond to the genomes considered: *E. coli* MG1655, *Haemophilus influenzae* strain NML-Hia-1, *Citrobacter freundii* strain 705SK3, *Escherichia coli* O157:H7 strain JEONG-1266, *Acinetobacter baumannii* strain K09-14, *Staphylococcus aureus* strain Bmb9393, *Klebsiella pneumoniae* strain ATCC BAA-2146, which are represented by the red, dark blue, orange, dark red, gray, black, and purple curves, respectively.
