# Supplementary material for: Highly mismatch-tolerant homology testing by RecA could explain how homology length affects recombination
Source: PLoS One. 2023 Jul 13;18(7):e0288611. doi: 10.1371/journal.pone.0288611 (PMC10343044; doi:10.1371/journal.pone.0288611)
Supplement: S6 Fig — (DOCX) [file pone.0288611.s006.docx]

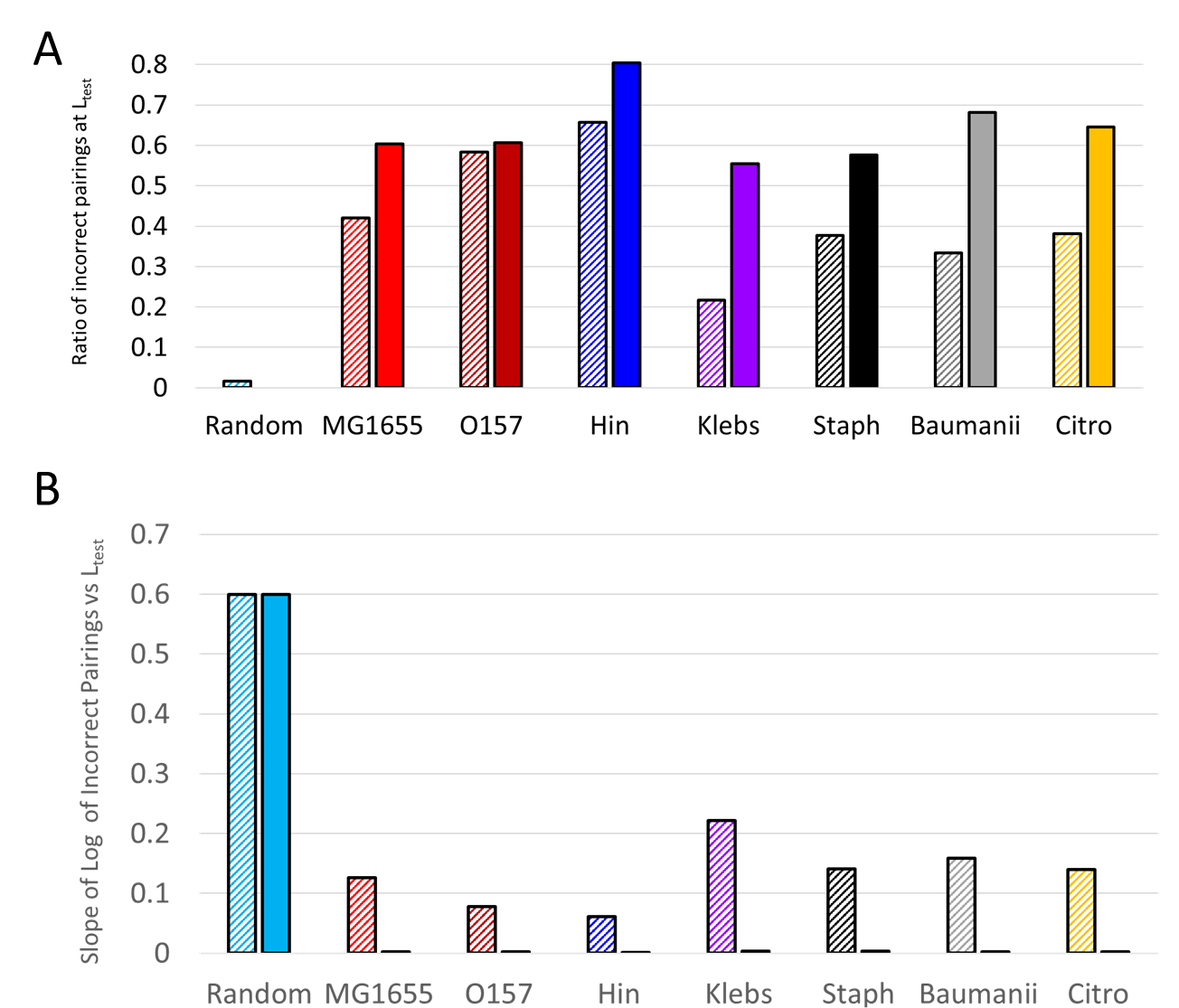


**S6 Fig. Saturation in the decrease in incorrect pairings as L_test_ increases. (A).** The bars with the striped fills indicate the ratio of the stringency for L_test_ = 17 to the stringency for L_test_ = 14. Smaller bars indicate that stringency increases more quickly with L_test_. The bars with solid fills indicate the ratio of the stringency for L_test_ = 99 to the stringency for L_test_ = 17. For all bacterial genomes, the solid bar is higher than the striped bar. **(B).** Same as A, but the bars represent the absolute value of the slope of the Log of the probability of an incorrect pairing as a function of L_test_. The slopes are always negative. For the random genome, the slopes are always the same for L_test_ ⪆ 14. In contrast, for the bacterial genomes the slopes from L_test_ = 17 to L_test_ = 99 are barely visible on the graph, indicating that the increase in accuracy with L_test_ strongly saturates when L_test_ ⪆ 17.
