# Supplementary material for: Highly mismatch-tolerant homology testing by RecA could explain how homology length affects recombination
Source: PLoS One. 2023 Jul 13;18(7):e0288611. doi: 10.1371/journal.pone.0288611 (PMC10343044; doi:10.1371/journal.pone.0288611)
Supplement: S7 Fig — (DOCX) [file pone.0288611.s007.docx]

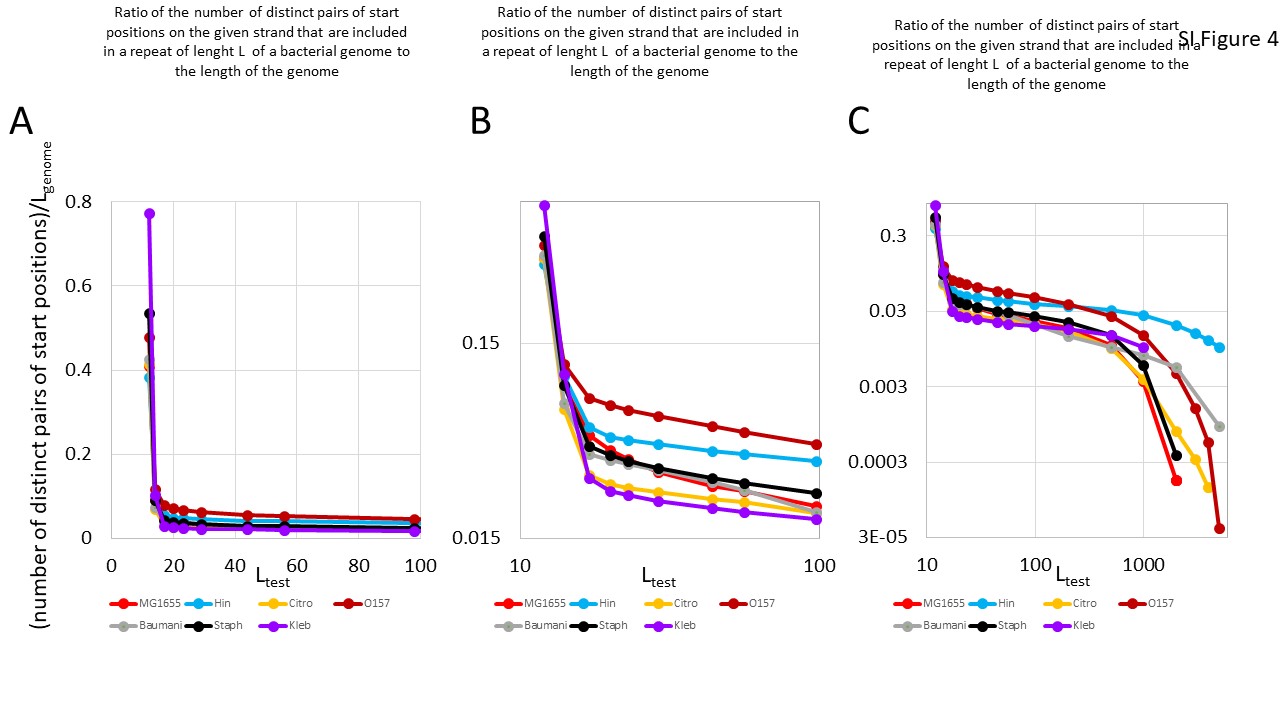


**S7 Fig.** **Ratio of the number of distinct pairs of starting locations in the given strands of bacterial genomes that share a repeat of length L to the genome length as a function of L.** **(A).** The color of the curve corresponds to the genome considered. *E. coli* MG1655, *Haemophilus influenzae* strain NML-Hia-1, *Citrobacter freundii* strain 705SK3, *E. coli* O157:H7 strain JEONG-1266, *Acinetobacter baumannii* strain K09-14, *Staphylococcus aureus* strain Bmb9393, *Klebsiella pneumoniae* strain ATCC BAA-2146 are represented by the red, blue, orange, dark red, gray, black, and purple curves, respectively. Both the x and y axis are linear. **(B).** Same but the x and y-axes are logarithmic. **(C).** Same as B but the x-axis is extended to 6000 bp.
