# Supplementary material for: Highly mismatch-tolerant homology testing by RecA could explain how homology length affects recombination
Source: PLoS One. 2023 Jul 13;18(7):e0288611. doi: 10.1371/journal.pone.0288611 (PMC10343044; doi:10.1371/journal.pone.0288611)
Supplement: S8 Fig — (DOCX) [file pone.0288611.s008.docx]

**
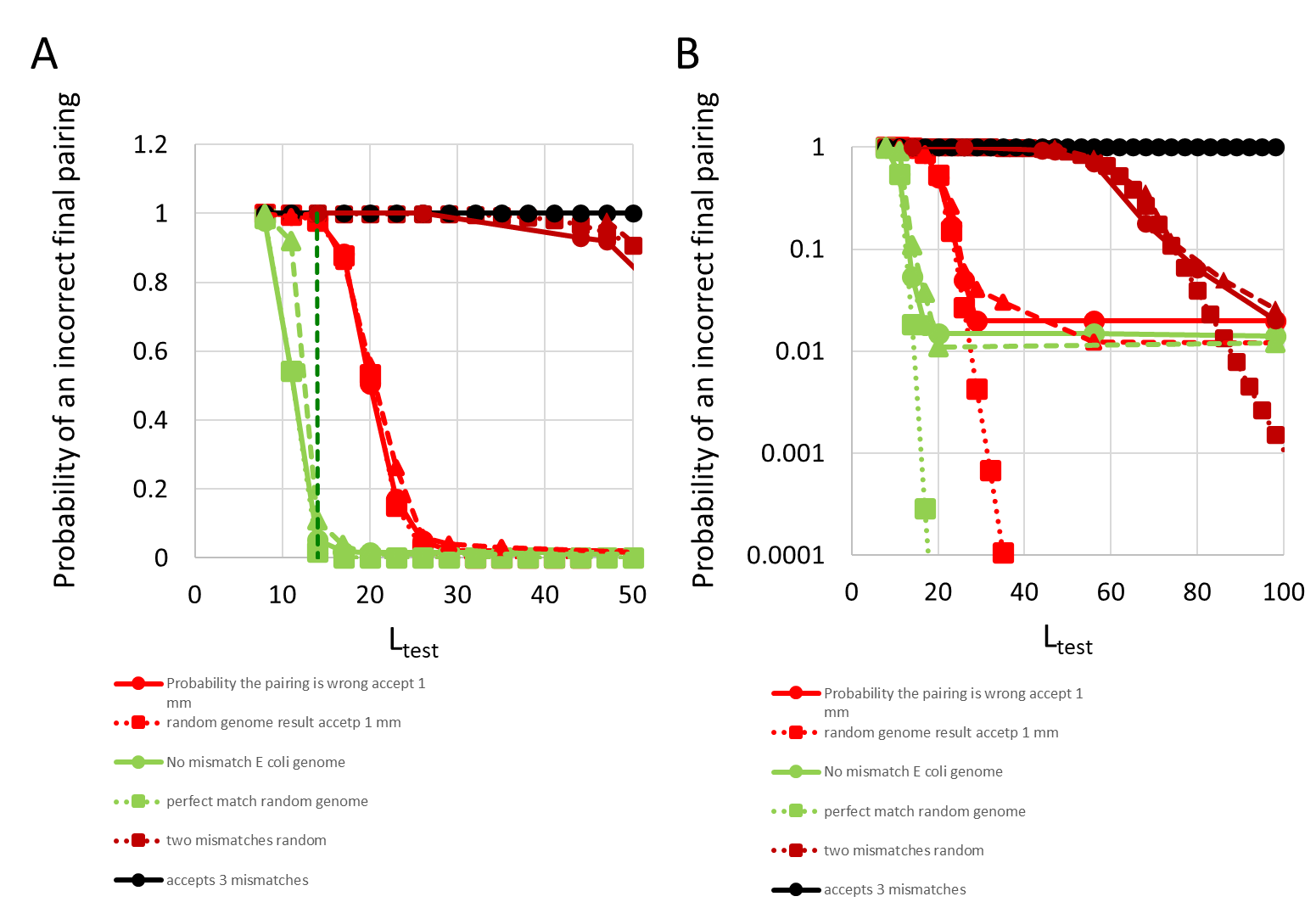
**

**S8 Fig.** **Probability that a DSB will result in an incorrect final pairing vs. L_test_ if invading strands all terminate in Chi sites.** (**A)**. Probability that a final pairing will be incorrect as a function of L_test_ and N_mismatch_. The light green lines show results for sparse homology testing that requires complete sequence matching. The bright red, dark red, and black lines with circular symbols indicate results for sparse homology testing when the 8-bp test that accepts one mismatch is followed by triplet tests that accept 1, 2, and 3 mismatches, respectively. The dark green vertical dashed line indicates L_test_ = 14. (**B)**. Same as A with a logarithmic y-axis.
