# Supplementary material for: Highly mismatch-tolerant homology testing by RecA could explain how homology length affects recombination
Source: PLoS One. 2023 Jul 13;18(7):e0288611. doi: 10.1371/journal.pone.0288611 (PMC10343044; doi:10.1371/journal.pone.0288611)
Supplement: S9 Fig — (DOCX) [file pone.0288611.s009.docx]

**
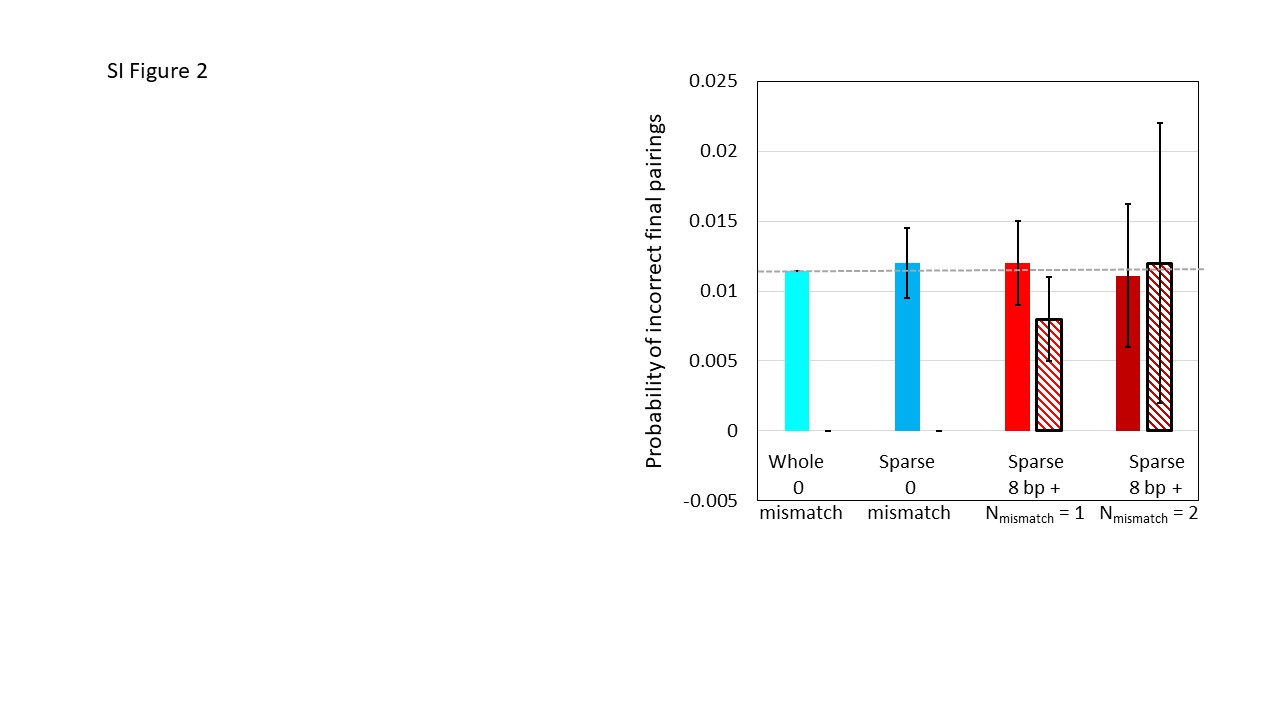
**

**S9 Fig. Predicted incorrect DSB repairs for L_test_ = 98.** Fraction of DSB in *E. coli* MG1655 that would be incorrect and contain no mismatches (solid bars) or incorrect and contain mismatches (striped bars). The cyan and blue bars show results of homology tests that accept no mismatches. The cyan bars show the exact result for the entire given strand in *E. coli* MG1655. The blue bars show the result for sparse sampling. The red and dark red bars show results for an 8 bp test that accept a mismatch with triplet testing that accepts N_mismatch_ = 1 or N_mismatch_ = 2 mismatches/triplets, respectively.
